# Supported implementation of tailored hospital fall prevention interventions: a protocol for the PROTECT stepped wedge type I hybrid effectiveness-implementation trial

**DOI:** 10.1136/bmjopen-2025-111744

**Published:** 2026-03-19

**Authors:** Charlotte McLennan, Leanne Hassett, Wendy Tilden, Vasi Naganathan, Abby Haynes, Matthew Jennings, Danielle Ni Chroinin, Bethan Richards, Andrew Hallahan, Raaj Kishore Biswas, Wing Kwok, Tamsin McVeigh, Erica Heppleston, Debra Jackson, Veethika Nayak, Sally Delaney, Kirsten Howard, Marina Pinheiro, Amanda Macpherson, Jenny Rayner, Anne-Marie Hill, Terry Haines, Catherine Sherrington

**Affiliations:** 1 Institute for Musculoskeletal Health, Sydney Local Health District, Camperdown, New South Wales, Australia; 2 School of Public Health, The University of Sydney Faculty of Medicine and Health, Sydney, New South Wales, Australia; 3 Sydney Health Partners Implementation Science Academy, Sydney, New South Wales, Australia; 4 Sydney Local Health District, Camperdown, New South Wales, Australia; 5 Concord Clinical School, Faculty of Medicine and Health, University of Sydney, Sydney, New South Wales, Australia; 6 Centre for Education and Research on Ageing, Department of Geriatric Medicine, Concord Hospital, Concord Repatriation General Hospital, Concord, New South Wales, Australia; 7 Physiotherapy, South Western Sydney Local Health District, Liverpool, New South Wales, Australia; 8 Aged Care, Liverpool Hospital, Liverpool, New South Wales, Australia; 9 SWS Clinical School, University of New South Wales, Sydney, New South Wales, Australia; 10 Institute for Musculoskeletal Health, Sydney School of Public Health, The University of Sydney, Sydney, New South Wales, Australia; 11 School of Health Sciences, The University of Sydney Faculty of Medicine and Health, Sydney, New South Wales, Australia; 12 Clinical Research Centre, Sydney Local Health District, Camperdown, New South Wales, Australia; 13 Susan Wakil School of Nursing and Midwifery, The University of Sydney Faculty of Medicine and Health, Sydney, New South Wales, Australia; 14 School of Public Health, University of Sydney, Sydney, New South Wales, Australia; 15 South Western Sydney Local Health District, Liverpool, New South Wales, Australia; 16 Consumer Investigator, Sydney, New South Wales, Australia; 17 School of Allied Health, The University of Western Australia, Perth, Western Australia, Australia; 18 WA Centre for Healthy Ageing, The University of Western Australia, Perth, Western Australia, Australia; 19 Monash University School of Primary and Allied Health Care, Frankston, Victoria, Australia

**Keywords:** GERIATRIC MEDICINE, Health Services, Hospitals, Clinical Trial

## Abstract

**Introduction:**

Patient falls in hospitals lead to patient harm, staff distress and economic burden on health systems. There are few strategies with robust evidence demonstrating benefit for the prevention of falls, especially in acute hospital settings. Education and multicomponent fall prevention approaches are promising. Rigorous systematic measurement of implementation has been lacking in most hospital fall prevention trials. This paper describes the protocol for a trial that will evaluate the impact of supported implementation of tailored multicomponent fall prevention interventions on patient falls in hospital.

**Methods and analysis:**

A stepped-wedge hybrid type I effectiveness implementation cluster randomised trial will be conducted. Twelve inpatient wards across four metropolitan hospitals will be enrolled in the trial, clustered into groups of four and randomised to commence the intervention at one of three time periods. Patients and ward staff will be recruited to complete pre-implementation surveys, which, combined with analysis of routinely collected local falls data and staff brainstorming, will inform tailored multicomponent fall prevention interventions for each ward. Wards will receive quality improvement training, clinical facilitation and staff education for at least 4 months to support implementation of their fall prevention interventions. The primary outcome—rate of falls—will be measured using routinely collected hospital falls data from the incident management system and medical records. Pre-implementation and post-implementation patient and staff surveys, qualitative interviews and bedside audits will measure secondary effectiveness and implementation outcomes. Healthcare utilisation from hospital data will inform the cost-effectiveness analysis.

**Ethics and dissemination:**

The Sydney Local Health District Human Research Ethics Committee (RPAH Zone) approved this trial (protocol number X24-0087 and 2024/ETH00583). The trial is registered with the Australian and New Zealand Clinical Trials Registry (ACTRN12624000896572). Data collection commenced in October 2024, due for completion in May 2026. Results will be published in reputable international journals and presented at relevant conferences.

**Trial registration number:**

Australian and New Zealand Clinical Trials Registry (ACTRN12624000896572).

**STRENGTHS AND LIMITATIONS OF THIS STUDY**

- This is a large multicentre stepped-wedge cluster randomised trial including a mix of acute and subacute clinical specialities across 12 wards in four hospitals.
- The evaluation of effectiveness, implementation and economic outcomes will provide much-needed robust evidence on the effect of tailored fall prevention interventions and implementation strategies; important evidence considering the global ageing population.
- Falls and fall injury outcomes will be measured via staff-reported data. There is the possibility of increased reporting of falls on wards once they cross over from control periods into intervention periods due to heightened awareness of falls.
- There are multiple, complex factors influencing hospital falls, and it is not possible to address all of these in this trial (eg, the impact of external contextual factors).

## Introduction

 Falls in hospitals remain a common and costly problem for health systems, health staff and patients and their families.^1 2^ They can lead to significant physical injuries and death, especially in older people.^3 4^ Even without injuries, hospital falls can reduce confidence, activity levels and independence, increase length of stay and alter discharge destinations.^5^ No single approach or intervention has proved a solution for this complex problem across broad acute and subacute hospital patient populations.^6^,^8^ This is reflective of the diversity and complexity of patients and contexts in which falls occur.

The recent World Falls Guidelines highlight the need for hospital fall prevention strategies that are tailored to specific environments and personalised for individual patients.^4^ Tailored multicomponent fall prevention interventions, especially those involving patient education, show a promising impact on hospital falls.^9 10^ However, individualised patient education is time-consuming and unlikely to be cost-effective on acute wards with rapid through-put and a lower total proportion of people who fall in hospital.^11^ Large trials of patient education interventions have also had inconsistent results in patients with cognitive impairment.^9 12 13^ Among hospital inpatients aged 65 and over, 29%–38% are estimated to have cognitive impairment.^14^ One study across a large Australian metropolitan hospital found 43% of inpatients over 65 demonstrated cognitive impairment.^15^ The prevalence of inpatients with cognitive impairment is likely to grow with the growing proportion of hospital admissions by older people.^16^

The implementation of tailored multicomponent fall prevention interventions into routine practice can be challenging and variable.^17 18^ Targeted implementation strategies that are rigorously designed and address barriers to implementation are required to support this process.^2 19 20^ The research team’s prior work explored barriers and facilitators to delivery of multicomponent hospital fall prevention interventions in four local hospital wards, similar in context to the wards planned for this trial.^18 21^ The barriers and facilitators were mapped to the COM-B model of behaviour change to identify suitable implementation strategies targeting ward level capabilities, opportunities and motivation.^22^ The strategies were then tested in a feasibility study in those wards and further refined based on the results. Subsequent mapping (included in online supplemental material 1) identified four core implementation strategies that, when tailored in collaboration with local ward staff to their local context, may support effectiveness of the tailored multicomponent fall interventions in this trial. These include quality improvement training; clinical facilitation; ongoing training and education and building a coalition. These implementation strategies are intended to build workforce capacity for developing and sustaining change.^23^

Economic evaluations are crucial to informing the cost-effectiveness, or value for money, of hospital fall prevention interventions and implementation strategies, thus providing critical evidence for decision-making in policy and practice, especially at scale.^24^ Guidelines for economic evaluation of fall prevention strategies have been available since 2011.^25^ Despite this, few trials of interventions to reduce falls in hospital settings incorporate economic evaluations.^7^

This paper describes the protocol for a stepped-wedge trial that will evaluate whether supported implementation (quality improvement training, clinical facilitation, ongoing training and education and building a coalition) of tailored ward-based fall prevention interventions reduces patient falls in hospitals. The trial will also evaluate the impact of supported implementation of tailored ward-based multi-component fall prevention interventions on a range of secondary effectiveness outcomes, and implementation outcomes and determinants. The cost-effectiveness of the programme from a hospital perspective will also be evaluated.

## Methods

### Study design, randomisation, setting and participants

This study is a stepped wedge pragmatic hybrid type I effectiveness-implementation cluster randomised trial.^26 27^ It has a nested economic evaluation. The trial design involves the random and sequential crossover of clusters of hospital wards from control to intervention units until all units are exposed to the intervention. This study design was chosen as it involves recruitment and intervention implementation at a ward level which, in a context of an intervention that has ward-level impacts, can lead to less bias in the results.^26^ The pragmatic design allows enrolment of diverse patient and staff populations and embeds the research within everyday clinical workflows, enhancing the external validity, generalisability and scalability of the findings.^28 29^ The design also allows for the implementation support to be delivered at staggered times, making delivery more feasible.^30^ This protocol follows the standard protocol items: recommendations for interventional trials statement (online supplemental material 2).^31^ The trial is registered with the Australian and New Zealand Clinical Trials Registry - trial registration number ACTRN12624000896572.^32^

**Figure 1:**
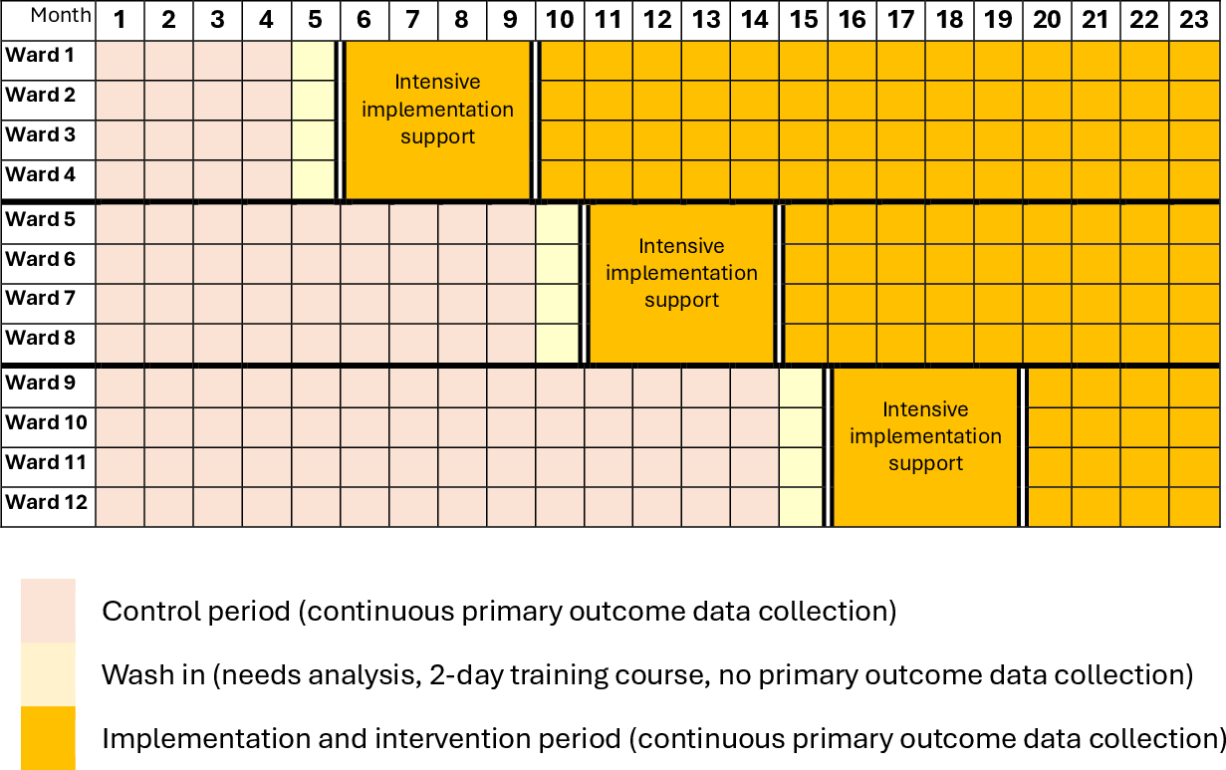
Stepped-wedge trial design.

**Figure 2:**
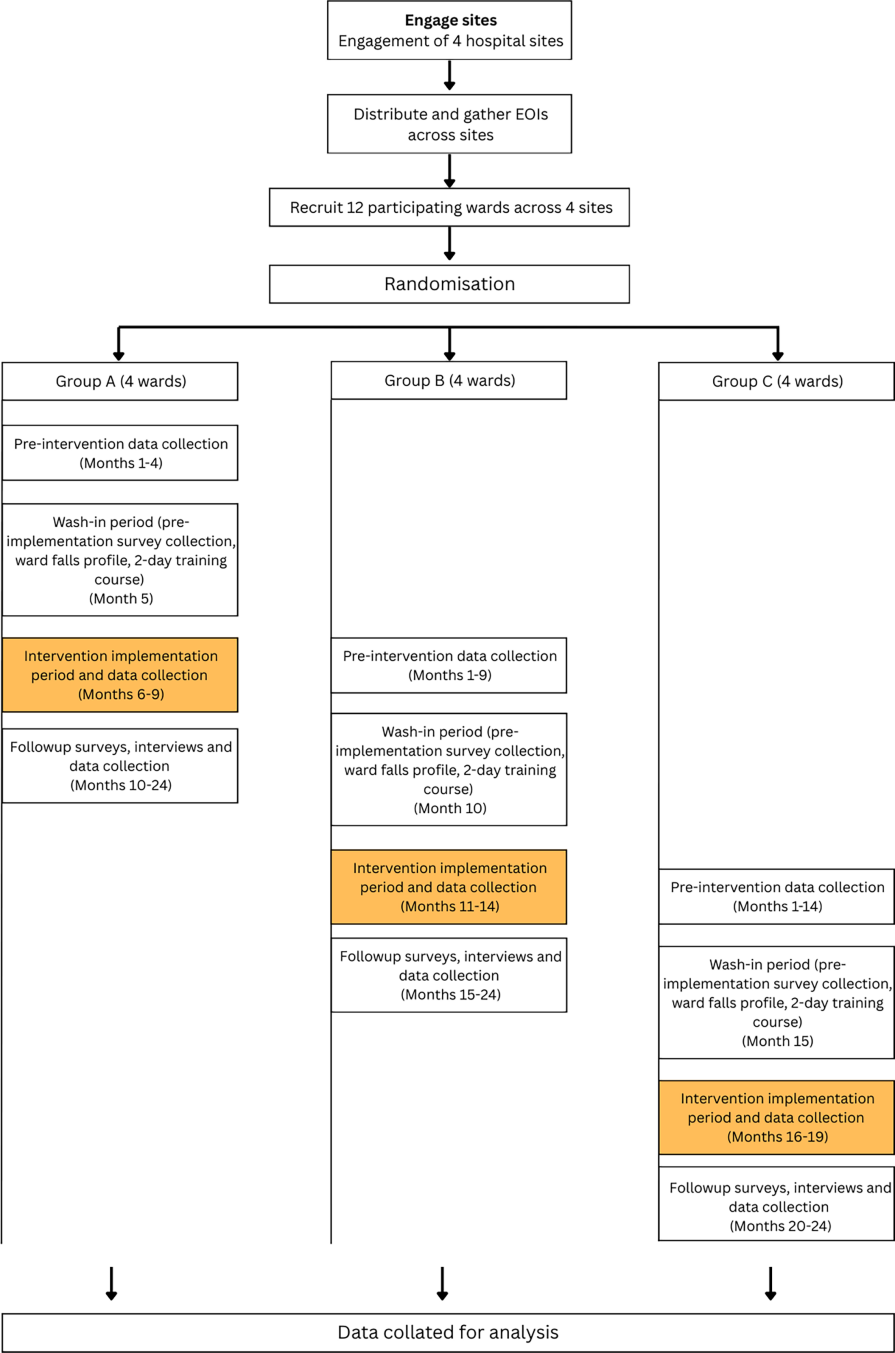
Study flowchart. EOI, Expression of Interest.

The trial will be conducted across 12 inpatient hospital wards across four public hospitals in Sydney, Australia. The participating wards will be a mix of clinical specialities and include acute and sub-acute care wards. Wards will be grouped into clusters of four based on geographic proximity. This clustering approach will ensure feasibility of intervention delivery while also minimising risk of contamination bias and promoting coherence with ward and organisational processes.^33 34^ Wards will be randomised using a secure computer-based system to commence the implementation support at one of three 4-month time periods. Randomisation will be led by a member of the study team (RB) blinded to the ward clusters and who will not be involved in recruitment, implementation support or intervention delivery. The majority of the implementation strategies will be delivered to ward staff during the 4-month (17 week) intensive implementation support periods. There will be a wash-in period for 1 month prior to the intensive implementation period of each cluster to allow for collation of data to inform ward fall profiles and for some initial tailoring of implementation strategies and the multicomponent falls prevention intervention. Wards will commence improvement science training in this wash-in period to ensure they have baseline knowledge to optimise support from the clinical facilitator at the start of the intensive implementation support period. Primary outcome data will not be collected during the wash-in period as this period is not clearly control phase or clearly intervention phase data.^35^ Figure 1 indicates the study design and figure 2 provides the study flow chart.

Staff from nursing, medical, physiotherapy, occupational therapy, dietetics, speech pathology, social work, pharmacy, podiatry, orthotics, diversional therapy, administration and environmental services disciplines working on the participating wards, and inpatients and their family members on the participating wards, will be invited to complete pre-implementation and post-implementation surveys. Given the high turnover of inpatients and staff in many wards and to keep survey responses anonymous, participants in the pre-implementation and post-implementation surveys do not need to be the same. Staff, inpatients and their families will also be invited to participate in post-implementation interviews.

Inclusion and exclusion criteria for the various aspects of the study are provided in table 1.

**Table 1:** Inclusion and exclusion criteria for components of the study

Participants	Inclusion criteria	Exclusion criteria
Hospital wards	Inpatient hospital wards at participating hospitals. Support for nursing and other involved discipline executives to participate in the programme.	Emergency departments. Intensive care units. Paediatric and neonatal wards. Wards currently participating in another research trial with patient falls as a listed outcome. Outpatient settings.
Staff pre-implementation survey participants	Employed by participating hospitals. Work in nursing, medical, physiotherapy, occupational therapy, dietetics, speech pathology, social work, pharmacy, podiatry, orthotics, diversional therapy, administrative or environmental services teams on a ward enrolled to participate in the intervention.	Nil.
Staff post-implementation survey and interview participants	Employed by participating hospitals. Work in nursing, medical, physiotherapy, occupational therapy, dietetics, speech pathology, social work, pharmacy, podiatry, orthotics, diversional therapy, administrative or environmental services teams on a ward that participated in the intervention.	Has worked on the participating ward for <1 week.
Patient and family member/carer pre-implementation and post-implementation survey participants	Admitted inpatient or family member/carer of admitted inpatient on a participating ward at a participating hospital. Deemed cognitively and medically well enough to participate in a survey as per Nurse Unit Manager of the ward’s clinical judgement. Aged 18 years of age and over.	Inpatient on a ward not participating in intervention. Deemed not cognitively and/or medically well enough to participate in a survey as per the Nurse Unit Manager of the ward’s clinical judgement.
Patient and patient family member/carer interview participants	Admitted inpatient or family member/carer of admitted inpatient on a participating ward at a participating hospital. Deemed cognitively and medically well enough to participate in an interview as per Nurse Unit Manager of the ward’s clinical judgement. Aged 18 years of age and over.	Inpatients or visitors on wards not participating in intervention. Deemed not cognitively and/or medically well enough to participate in an interview as per the Nurse Unit Manager of the ward’s clinical judgement. Has been on ward for <24 hours.

### Participant recruitment

#### Recruitment of participating wards

Twelve eligible inpatient hospital wards will be recruited to participate in the PROTECT Fall prevention programme and trial via an invitation to voluntarily submit an expression of interest (EOI). The EOI invitation and a participant information statement (PIS) will be circulated via e-mail by a district Fall Prevention and Management Coordinator to the Nurse Unit Managers and Clinical Nurse Educators of the wards of participating facilities. Wards have 2 weeks to decide if they wish to submit an EOI to participate. Ward teams will be provided with the opportunity to ask questions about the study by contacting the research team and by invitations to attend online drop-in information sessions.

If a ward wishes to participate in the study, they will be asked to return a completed EOI form to the study team. The returned EOIs will be screened to ensure they meet inclusion and exclusion criteria. If more than 12 EOIs are received, a selection of wards that optimise diversity of case mix and facility location will be selected. Wards that submit an EOI with a multidisciplinary team will be prioritised. The Nurse Unit Manager of eligible wards will be invited to provide informed consent for their ward to participate in the trial.

We have received a waiver of consent to collect patient outcome data (via routinely collected hospital data) for inpatients admitted to the participating wards during the data collection period.

#### Recruitment of staff pre-implementation and post-implementation survey participants and staff post-implementation interview participants

Staff on the participating wards will be recruited to participate in pre-implementation and post-implementation surveys and post-implementation interviews. Staff will be invited to participate via flyers with a URL link and a QR code that take potential participants to the relevant PIS. Following review of the survey PIS, staff can consent to participating, which will then take them to the survey. Staff interested in participating in an interview can contact the study team directly via details on the PIS and flyer. Verbal or written consent will take place prior to the interviews.

#### Recruitment of patient and family member pre-implementation and post-implementation survey and post-implementation interview participants

Potential patient and family member survey and interview participants will be screened for suitability to participate in a survey or interview by clinical judgement of the Nurse Unit Manager of each participating ward. Screening will consider whether participants have sufficient cognitive capability and are medically stable enough to complete the survey and/or participate in a short interview. The Nurse Unit Manager will notify the study team of patients deemed suitable to participate. The Nurse Unit Manager will also notify the study team if a patient has a family member that is suitable to approach in the instances of patients who are not cognitively suitable to participate. A member of the study team will then provide suitable potential participants with a flyer and PIS for the survey or interview. For the survey, participants can select to access the survey via a QR code or request a paper version of the survey for completion. Submission of the electronic or paper survey will indicate their consent to participate in the survey. For the interview, patients and family members will be given at least 12 hours to consider participation after receiving the PIS. A member of the study team will return to the patient’s bedside following this minimum review time and if the patient or family member wishes to participate in an interview, a time for this will be arranged. Written consent will take place prior to the interviews.

Patients with limited English can be invited to participate and consent via use of the culturally and linguistically diverse assist app^36^ and tablet device available on participating hospital wards. An interpreter can be booked via the Health District Interpreter service to translate for survey and interview completion.

### Sample size

Our design will provide 80% power to detect a 25% lower fall rate for intervention compared with the control phases (IRR=0.75), using a significant level of α at 0.05. Sample size calculations used the method described by Hemming and Taljaard^37^ with coefficients from previous studies^6 8^ and data from our partner organisations. To determine the appropriate sample size for the complete stepped-wedge design, we used the Stata function “steppedwedge”^38^ following the Hussey and Hughes approach,^39^ and conservatively assumed an intra-cluster correlation of 0.007.

The primary outcome is the falls rate (falls per 1000 bed days). Based on partner organisations’ monitoring data, we determined that 30 beds per ward for three steps and four clusters per sequence will result in 14 400 bed days in four wards for 120 days (3 months), providing >80% power.^38^,^40^ An estimated 5808 patients are expected to be admitted to the study wards during the study period.

Sample size calculations for the pre-implementation and post-implementation surveys were based on paired t-tests using survey data from our feasibility study. Assuming a SD of 0.8, the proposed sample sizes will provide 80% power to detect a mean change of 0.5 points on the Likert scale, as measured by survey questions.

### Blinding

Ward staff participants will not be blinded as they will be aware they are receiving the implementation support. It is possible that patients whose routinely collected data informs the primary outcome are blinded to their participation as implementation is at a staff level and a waiver of consent for patient participation has been granted. However, this is not rigorous enough to formally refer to patient participants as blinded.

Persons assessing falls will be blinded to the intervention periods. Data analysts will be blinded for the primary data analysis.

### Intervention

The clinical intervention to be implemented into practice is informed by a Fall Prevention Strategy which was released by the district health authority that governs the participating hospitals.^41^ The strategy emphasises tailored approaches to hospital fall prevention. It encourages interventions that centre on ensuring people at risk of falling receive individualised interventions which meet their needs and incorporate principles of comprehensive care.^42^ The strategy also advises using ward-level data to guide improvement.

Participating wards are required to establish a local project team of clinical staff to co-design and lead the implementation of tailored, multicomponent fall prevention interventions on their ward. Ward teams are required to have the written support of the relevant Director of Nursing to participate in the programme. In cases of physiotherapy, occupational therapy, dietetics, speech pathology, social work, pharmacy, podiatry, orthotics and/or diversional therapy involvement, in the project team, verbal support from their respective manager is required. This combination of ‘bottom-up’ engagement in co-design and explicit ‘top-down’ leadership advocacy is intended to motivate ward teams and support them to embed local interventions into routine practice. The selection and co-design of specific interventions is informed by each ward’s local fall incident data and the ward’s unique patient, organisational and environmental characteristics and follows the Agency for Clinical Innovation’s codesign framework.^43^ Ward teams attend workshops during the improvement science training and on the ward during the intensive implementation period where they brainstorm key causes of falls on their ward and potential management strategies (informed by staff and patient surveys, evidence updates and staff experiences). This includes consideration of the cultural and linguistic backgrounds of the patient cohorts on their ward. The ward teams work with an experienced clinical facilitator to prioritise, select and tailor interventions based on their anticipated impact and achievability. The number, timing and duration of workshops is dependent on ward needs.

During the preintervention control phases, wards continue usual care including participation in any facility-based or district-wide initiatives to support the health district’s Fall Prevention Strategy. This is appropriate as our trial is testing additional support for the implementation of tailored interventions informed by the Strategy.

### Implementation strategies

The multi-faceted implementation approach is delivered to ward staff/clinicians to support them to implement their selected tailored fall prevention interventions into their local ward. The approach involves core implementation strategies of quality improvement training, clinical facilitation, ongoing staff education and training and the enduring building and maintenance of a coalition. Details of the strategies are presented in table 2 following Proctor’s *et al*’s recommendations for reporting implementation strategies.^44^ Each core strategy may branch into or incorporate additional strategies defined as per the expert recommendations for implementing change and compilation of implementation strategies.^45^ A more detailed overview of the implementation strategies is provided in online supplemental material 3. Additional implementation strategies may be created to meet individual ward needs based on their pre-implementation needs profile and selected tailored falls prevention interventions.

**Table 2:** Implementation strategies

**Core implementation strategy: training (quality improvement)** **Action:** a pre-existing **i**mprovement science training course is run by the Local Health District’s education department. Quality improvement is a system of iterative review and refinement aiming to produce positive changes to optimise local and organisational outcomes.^61^ Training topics include: using diagnostic data to inform change ideas; assessing and implementing change ideas’ via Plan-Do-See-Act cycles and assessing, spreading and sustaining improvement. The course will use mixed modes of training, including presentations, workshops and interactive activities. The course will have 2 hours of additional hospital fall prevention evidence summary content added for the trial. **Justification:** research suggests quality improvement training is acceptable and feasible in supporting the implementation of multicomponent fall prevention interventions.^21 62^ Face-to-face training is perceived to be more beneficial than online training.^21^				
**Actor**	**Target(s**)	**Temporality**	**Dose**	**Outcome**
Training run by clinicians who are experts in quality improvement and fall prevention. The primary trainer is an Educator working for the health district’s education department.	A minimum of two clinicians from each participating ward who will be leading their team’s implementation of multicomponent fall prevention interventions.	Staff attend the course within the 2 week period prior to the commencement of the intensive clinical facilitation support.	Once-off, 2 day face-to-face course.	Adoption, reach, dose, sustainability.
**Core implementation strategy: facilitation** **Action**: a clinical facilitator provides ongoing training and support to clinical staff on improvement science methodology following their 2 day training course. Clinical facilitation is underpinned by theoretical models of behaviour change and involves an experienced clinician providing mentorship, guidance and support to clinical teams. Training and education may include topics such as using available data to inform and evaluate change; practical understanding of how to adapt ways of working to reflect their distinct ward/area setting, work culture, patient cohort and specific local challenges and implementing and managing change. The clinical facilitator can also provide the tailored ongoing fall prevention education and training described in the core implementation strategy below.^63^ **Justification:** clinical facilitation was successfully used to support multicomponent fall prevention intervention in a multi-site quality improvement project.^62^ The team’s prior research informing this study found clinical facilitation is acceptable and feasible in supporting the implementation of multicomponent fall prevention interventions.^23^				
**Actor**	**Target(s**)	**Temporality**	**Dose**	**Outcome**
The clinical facilitator is a full-time, trial-funded position filled by a senior Nurse Specialist. Their workload is distributed across the four participating wards at a time.	Staff on participating wards implementing multi-component fall prevention interventions.	Clinical facilitation commences in week one of the participating wards’ 17 week intensive implementation support period.	The clinical facilitator will meet face-to-face with the participating teams for at least 30 min, once a week during the 17 week intensive implementation period and is available for access at other times on an as-needs basis. Teams then move to a minimum of 4 months of 30 mins clinical facilitation per month (with additional support on request).	Adoption, reach, dose, fidelity, sustainability.
**Core implementation strategy: ongoing education and training** **Action:** tailored education about risks for and consequences of falling in hospital and training sessions on the practical delivery of ward-selected fall prevention strategies. Using adult learning techniques, staff education and training can increase staff engagement, skills, efficacy, confidence and motivation.^64 65^ **Justification:** staff education and training is an integral component of the delivery of effective fall prevention interventions in large trials in hospital settings.^9 66^ It was identified as a facilitator to multicomponent fall prevention in the team’s prior research informing this study.^18 21^				
**Actor**	**Target(s**)	**Temporality**	**Dose**	**Outcome**
Sessions are run by physiotherapy and nursing members of the study team experienced in hospital fall prevention and by the clinical facilitator.	Staff on participating wards implementing multi-component fall prevention interventions.	Education and training sessions occur during the intensive implementation phase.	The specific content, number and timing of sessions is informed by ward staff needs and preferences identified in the staff pre-implementation surveys, in discussion with ward staff and in testing their selected small local changes.	Adoption, reach, dose, fidelity.
**Core implementation strategy: build a coalition** **Action**: internal and external stakeholders are engaged to optimise the implementation effort. Justification: the research team’s relationships with broad stakeholder groups were crucial to the work informing this study.^18 21^ The implementation science field identifies a need for stakeholder engagement to optimise research and healthcare delivery.^67^				
**Actor**	**Target(s**)	**Temporality**	**Dose**	**Outcome**
The research team intentionally maps and engages stakeholders.	Internal and external stakeholders who will be involved, influence and/or impact the intervention and implementation strategies. Cohorts include but are not limited to academics, health executives and managers, clinicians, hospital staff, patients and families.	Commenced in months prior to study and in previous work informing the study. Continues throughout and after the study period.	The specific mode, number and timing of stakeholder interactions is dependent on stakeholder capacity and project needs.	Adoption, reach, dose, fidelity, sustainability.

### Outcomes

#### Primary effectiveness outcome

##### Rate of falls

Routinely collected data from the wards will be used to compare fall rates in intervention versus control periods, expressed as falls per 1000 patient bed days. The accepted definition of a fall as an unintended event where the participant comes to rest on the ground, floor or lower level, as a result of a loss of balance will be used.^46 47^ Near misses will not be included. Data will be extracted from an incident management system (where ward staff are mandated to report all falls and near misses) and coded hospital datasets from medical records to increase the validity of this measure. Falls will be adjudicated by staff masked to intervention timing.

### Secondary effectiveness outcomes

#### Rate of injurious falls and unwitnessed falls

Data on injuries associated with falls and unwitnessed falls will be extracted from hospital records and incident reports and adjudicated by staff masked to intervention period. Data will be extracted on (a) Documented injury, (b) Serious injury (harm score 1–2, eg, fractures, intracranial injuries) and (c) Falls that were not witnessed by a staff member. Data will be reported as the rate of injurious falls and the rate of unwitnessed falls per 1000 patient bed days.

#### Incidence of hospital-acquired delirium and hospital-acquired pressure injuries

Data on hospital-acquired delirium and pressure injuries will be extracted from hospital records and incident reports. Multicomponent, tailored interventions such as those delivered in this trial may improve the care for older people in hospital more broadly, which may be reflected in these related outcomes.

#### Patient length of stay

Data on patient length of stay on participating wards will be extracted from hospital records to monitor that the intervention is not associated with increased length of stay.

#### Staff professional fulfilment

Data on staff professional fulfilment will be collected via staff surveys and qualitative interviews informed by validated tools.

#### Patient mobility patterns

This will be informed by a staff survey question 4 months postintervention implementation and by qualitative data from staff and patient semi-structured interviews.

### Implementation outcomes and determinants

Implementation outcomes and determinants will be collected for the tailored fall prevention interventions as well as the implementation strategies. These are outlined in table 3 below, which also summarises the evaluation methods. Methods include quantitative surveys and qualitative interviews with key stakeholders to guide lasting implementation and scale-up,^46 48 49^ as well as address questions about barriers to and facilitators of the approach. Observations on wards, file audits and analysis of study records will also be used. Study records include field notes from study staff documenting timing, attendance and notable moods, dynamics and reflections of meetings and education sessions with participating wards. These measures have been informed by McKay’s implementation framework.^49^

**Table 3:** Overview of implementation measures

	Implementation of tailored ward-based fall prevention interventions	Measurement tool	Delivery of multi-faceted implementation strategy	Measurement tool
Implementation outcomes				
Adoption	Proportion and representativeness of participating ward staff that deliver tailored fall prevention intervention.	Ward-specific audits. Staff surveys.	Proportion and representativeness of the sites that use implementation strategies.	Study records. Staff surveys.
Reach	Proportion of patients provided with fall prevention interventions.	Ward-specific audits. Staff surveys. Patient surveys and interviews.	Proportion of eligible wards who submitted EOIs.	Study records.
Dose	Extent of delivery of selected intervention components.	Ward-specific audits. Staff surveys and interviews. Patient surveys and interviews.	Hours per clinical team of delivered multi-faceted implementation strategies.	Study records/fieldwork notes.
Fidelity	Extent to which selected fall prevention interventions are implemented.	Staff surveys and interviews.	Proportion of intended training, education and facilitation sessions provided to and attended by participating clinical teams.	Study records.
Sustainability	Intended ongoing delivery of fall prevention interventions after the 4-month clinical facilitation period.	Staff surveys and interviews.	Participation in peer support sessions after clinical facilitation period has ceased. Intended use of quality improvement skills in future work.	Study records. Staff surveys.
Implementation determinants				
Acceptability	Patient and staff views of interventions’ acceptability.	Staff surveys and interviews. Patient surveys and interviews.	Staff and manager impressions of the acceptability of the implementation strategies.	Staff surveys and interviews.
Feasibility	Perceptions among the delivery team that the intervention can be successfully used or carried out within the given setting.	Study records.	Perceptions among the delivery team that the implementation strategies can be successfully used or carried out within the given setting.	Study records.
Self-efficacy	Clinician confidence applying tailored fall prevention interventions.	Staff surveys and interviews.	Clinician confidence applying quality improvement methods.	Staff surveys and interviews.
Appropriateness	Extent to which the tailored fall prevention interventions fit with ward needs.	Staff surveys and interviews. Patient surveys and interviews.	Extent to which the implementation strategies fit with ward needs.	Staff surveys and interviews. Study records.
Satisfaction	Staff and patients’ satisfaction with the impact of the tailored fall prevention strategies.	Staff surveys and interviews. Patient surveys and interviews.	Staff satisfaction with the impact of the tailored fall prevention strategies.	Staff surveys and interviews. Patient surveys and interviews.

EOIs, Expression of Interests.

### Data collection methods

#### Effectiveness data

Routinely collected medical record data (including hospital coded data) incident management system data and quality audit reporting system data will be extracted for participating wards in the specified timeframes. These data are collected by the health facilities using routine methods, as required by hospital accreditation agencies. Data will be extracted for 5 months prior to the first cluster’s intervention implementation and for nineteen months following this. Primary outcome data will not be collected during the wash-in period. Figure 2 provides an overview of the study periods.

#### Implementation data

Implementation level data will be collected pre-implementation and post-implementation to measure implementation outcomes and determinants. This will be collected via:

- Staff pre-implementation and post-implementation anonymous surveys.
- Patient and patient family member pre-implementation and post-implementation anonymous surveys.
- Staff and patient/family interviews.
- Ward-specific pre-implementation and post-implementation audits (including observation and medical record review).
- Study records/fieldwork notes.

The staff pre-implementation and post-implementation surveys and interview guides (included in online supplemental material 4) have been piloted in the feasibility study that informed this trial.^21^ Changes to these surveys and interview guides were based on feedback from staff participants and study personnel in the pilot study (informally and formally through qualitative interviews). Additional questions have been added relevant to some implementation determinants and staff professional fulfilment, including from two validated survey tools: safety attitudes questionnaire^50^ and the professional fulfilment index.^51^ Implementation questions in staff and patient/family surveys are based on a recommended minimum dataset of indicators for implementation evaluation. These need to be specific to the intervention being implemented and thus cannot come directly from a validated tool. We have, however, been guided by survey and interview guide development literature.^52 53^ Free-text survey questions and specific questions in the interview guide intend to provide data informing the effectiveness and suitability of the individual implementation strategies and suggested future tailoring of these (see online supplemental material 4 for surveys and interview guides).

Ward-by-ward results of the pre-implementation survey data, pre-implementation ward audit data and pre-implementation incident management system data will inform an individual ward needs analysis and subsequent tailoring of the implementation strategy to meet these needs.

### Cost-effectiveness data collection methods

#### Health utilisation and intervention and implementation cost data

Intervention and implementation cost data for the economic evaluation will be collected from trial-based records for the trial duration. Healthcare utilisation during the trial duration will be collected from routinely collected coded hospital data. Healthcare utilisation will be valued using local or national costs as appropriate and all costs will be valued in Australian dollars. Discounting will not be applied, as the time horizon will be limited to the trial duration.

### Harms

Unexpected and related serious adverse events (URSAEs) perceived to be related to the trial intervention will be reported by the Nurse Unit Manager of the participating ward Manager (who will be educated about this procedure at the time of consent) to a member of the study team. URSAEs will be reported to the independent Data Safety Monitoring Board, as well as to the approving Human Research Ethics Committee (HREC), and amendments to the protocol will be considered as necessary.

### Statistical analysis

#### Effectiveness analysis

Statistical analysis will compare the rate of falls (primary outcome) and injurious falls and unwitnessed falls (secondary outcomes) between intervention and control time periods using a ward/time-period approach. The distribution of the falls will be examined as part of the model building, as these data are commonly skewed. If indicated, we will use negative binomial regression to estimate the difference in the rate of falls, expressed as an incidence rate ratio and 95% CI. Secondary analyses may use patient-level data and rates of falls per occupied bed days and will be clustered within ‘wards’. Secondary analyses using causal modelling may also be conducted to establish intervention effects in wards with greater uptake.^54^ Additionally, secondary analyses adjusting for covariates such as patient complexity may be undertaken. All analyses will be conducted while masked to group allocation and will use an intention-to-treat approach. Analyses will use Stata and R. Sensitivity analyses to adjust for any potential risks of bias that arise during the study may be conducted. The detailed statistical plan will be publicly available prior to the analysis commencing.

#### Survey analysis

All returned surveys that have at least one response will be included for analysis. Descriptive statistics (means, SD, change, counts and percentages) will be used to analyse survey data.

Quantitative implementation analysis of survey data will involve numerical description of quantitative implementation measures, presented as percentages or means and exploration with relative risks. Changes from pre-delivery to post-delivery of implementation strategies in self-reported staff and patient/family surveys will be evaluated using paired-sample t-tests. Univariate correlations will explore changes in these measures.

### Qualitative analysis

Audio recordings of semi-structured interviews will be transcribed and imported into NVivo software^55^ and analysed as a combined dataset using a qualitative descriptive approach.^56^ This aims to describe manager, frontline staff, patient and family experiences of the intervention and implementation strategies.^57^ This will be triangulated with content analysis of observational field notes to describe relevant contextual features including comparisons across wards which may help to explain outcomes. Iterative analysis consultation with the wider investigative team will be used to critique and refine emergent results.^58^

### Economic analysis

A trial-based economic evaluation will be conducted from a hospital perspective and the time horizon will be limited to the trial duration. The comparator will be usual care. All intervention implementation and delivery costs (from study records), including staff and equipment costs, and all health service utilisation costs (from hospital records) during the trial period (pre-implementation, implementation and follow-up periods) will be used for the analysis. We will calculate the incremental cost per fall prevented of the intervention periods compared with control periods.

Principles of economic evaluation alongside stepped wedge trials will be followed.^59^ Bootstrapping will estimate a distribution around costs and health outcomes and calculate the CIs around the incremental cost-effectiveness ratios. The results will be plotted on the cost-effectiveness plane. A cost-effectiveness acceptability curve will be created to explore the uncertainty around the probability that the intervention is cost-effective, when considering a decision maker’s willingness to pay for falls prevented. All analyses will be undertaken in Stata V.16 (Stata, College Station, TX, USA). The results of the cost-effectiveness analysis will be reported following the consolidated health economic evaluation reporting standards statement.^60^

### Trial monitoring and access to data

The trial investigators meet at least bimonthly to discuss the trial progress. The investigators will adhere to routine HREC reporting. A data safety and monitoring board (DSMB) has been established for this study with members independent to the study. The DSMB will convene in the event of an unanticipated serious adverse event that could be related to the intervention occurring during the trial. The DSMB charter can be accessed at request by contacting the research team.

All electronic study data will be stored on the sponsor’s secure password-protected servers, primarily the research electronic data capture server. Data linkage will be used for the coded hospital medical record and incident management system data to protect the privacy of individuals. Only de-identified data will be used for analysis. Strict privacy protection measures will be implemented to safeguard the confidentiality of individuals’ information. Data will only be accessed by approved members of the research team. All study data will be retained securely for a period of 15 years after study completion.

## Patient and public involvement

We have incorporated the perspectives of consumers and health professionals from study conception and continue to embed consumer involvement within the trial. Consumer Investigator JR is part of the research team and consults on the leadership and coordination of consumer involvement in this project. JR attends the bimonthly Investigator meetings and advises on the study outside of these meetings on an as-needs basis. A PROTECT Consumer Advisory Panel has been assembled. This advisory panel consists of patient and carer consumer representatives, and we meet with members every six to twelve months to review trial progress and provide input as needed. Consumers and health professionals have informed the design of the intervention and implementation strategies in this trial and have reviewed and edited participant materials including flyers, information statements and survey instruments.

The research team will meet with the Consumer Advisory Panel after results of the trial have been analysed. Input from the Consumer Advisory Panel as well as health professionals involved in the trial will be sought to help develop and disseminate plain language summaries of research results and findings.

## Ethics and dissemination

The Human Research Ethics Committee (RPAH Zone) of the Sydney Local Health District has approved this trial (protocol number X24-0087 and 2024/ETH00583). Updated procedures will only be implemented once they have been reviewed and approved by an Ethics Committee (or if applicable a governance office) for each site. Data collection commenced in October 2024 and is due for completion in May 2026. Results from the trial intend to be published in international journals and presented at relevant conferences.

## Supplementary material

10.1136/bmjopen-2025-111744 online supplemental file 1

10.1136/bmjopen-2025-111744 online supplemental file 2

10.1136/bmjopen-2025-111744 online supplemental file 3

10.1136/bmjopen-2025-111744 online supplemental file 4
