# Supplementary material for: Supported implementation of tailored hospital fall prevention interventions: a protocol for the PROTECT stepped wedge type I hybrid effectiveness-implementation trial
Source: BMJ Open. 2026 Mar 19;16(3):e111744. doi: 10.1136/bmjopen-2025-111744 (PMC13007140; doi:10.1136/bmjopen-2025-111744)
Supplement: online supplemental file 1 [file bmjopen-16-3-s001.pdf]

Supplementary material 1: Barriers identified in previous qualitative work and in stakeholder engagement mapped to COM-B model of behaviour change and associated implementation strategies

Desired behaviour: Ward staff deliver appropriate, tailored multicomponent fall prevention interventions within routine healthcare interactions

		**Barriers**	**Possible implementation strategies (selected by, and tailored to needs of, individual wards).<br>Overarching implementation strategy bracketed in bold.**
**Capability**	Physical	• Lack of knowledge of who is at risk of falling in hospital<br>• Lack of knowledge of how to reduce patient fall risk/ provide effective fall prevention interventions/ patient care<br>• Lack of access to published evidence to guide fall prevention interventions<br>• Inability to access and/or interpret local ward falls data to inform fall prevention interventions<br>• Lack of knowledge of how to interpret published and local data to inform fall prevention practice<br>• Unskilled in providing care to reduce patient fall risk e.g. situational awareness	• Ward based staff education by fall prevention experts (approx. 30 min sessions) and on the go by ward fall prevention team (potential topics: hospital fall prevention risks and mitigation, evidence-based fall prevention practice, human factors in aged care). **Ongoing education and training**<br>• 2026 Australian Hospital Fall Prevention Guidelines distributed to staff via e-mail and available on ward computer desktops. **Clinical facilitation**<br>• PROTECT Fall Prevention ward leadership team receive training on accessing and interpreting local falls data through incident system. **Quality improvement training**<br>• PROTECT Fall Prevention ward leadership team attend 2-day quality improvement training course. **Quality improvement training**<br>• Presentation of basic local data on a fall prevention board on the ward (e.g. Days since last fall poster. **Quality improvement training**<br>• Ward staff receive training on particular aspects of care to reduce patient fall risk e.g. situational awareness, safe patient mobility (topic as per needs of ward) **Ongoing education and training**<br>• Provision and discussion of summary of local incident data. Clinical facilitation Staff brainstorming sessions about drivers of falls. **Clinical facilitation**
	Psychological	• Difficulty setting SMART patient and ward goals relevant to fall prevention	• PROTECT Fall Prevention ward leadership team attend 2-day quality improvement training course. **Quality improvement training**

		• Insufficient clinical reasoning to tailor fall prevention care to individual patients<br>• Difficulty embedding fall prevention within routine practice and not as stand alone intervention<br>• Forgetting to provide fall prevention interventions within routine practice	• Ongoing clinical reasoning and goal setting training sessions facilitated by clinical facilitator for ward fall prevention team and/or ward staff. **Ongoing education and training**<br>• Ward based staff education by fall prevention experts and clinical facilitator (30 min sessions) and to staff on the go by PROTECT Fall Prevention ward leadership team (topics: SMART goals, clinical reasoning, embedding fall prevention into routine practice). **Ongoing education and training**<br>• Fall prevention promotion display on the ward including prompts for recommended staff fall prevention actions. **Clinical facilitation**<br>• Reminders re fall prevention program at routine ward meetings. **Ongoing education and training** Emphasis on celebration of fall prevention achievements (e.g. ward morning tea). **Clinical facilitation**
**Opportunity**	Social	• Fall prevention not usual practice within a discipline<br>• Fall prevention not usual practice on a ward<br>• Fall prevention not usual practice, supported or sponsored at a hospital and district Executive level<br>• Consistent and supportive leadership for fall prevention programs	• Engagement by PROTECT Fall Prevention Program team with multi-disciplinary stakeholders and multiple facility Executive prior to roll out of program (nursing, allied health, medical). **Build a coalition**<br>• Wards encouraged to have multi-disciplinary team members in their PROTECT Fall Prevention ward leadership team. **Build a coalition.**<br>• Wards to nominate clear lead for the project on their ward. **Quality improvement training.**<br>• Ward based staff education by fall prevention experts and clinical facilitator and on the go by ward fall prevention team (Example topics: evidence-based fall prevention practice, fall prevention success stories from similar setting/ disciplines). **Ongoing education and training**<br>• Discipline specific education sessions delivered by Fall Prevention Experts or Clinical Facilitator. **Ongoing education and training**<br>• SLHD Executive PROTECT Fall Prevention Program Steering Committee with multi-disciplinary and cross facility representation. **Build a coalition**<br>• Wards to engage an Executive sponsor/ champion for their involvement in the program (e.g. Facility Director of Nursing). **Build a coalition**

			• Executive level recognition of ward achievements during the program. **Build a coalition**<br>• Fall prevention promotion display on ward). **Ongoing education and training**<br>• Emphasis on celebration of fall prevention achievements (e.g. ward morning tea). **Clinical facilitation**<br>• Provision of some change management education to PROTECT Fall Prevention ward leadership team (during 2-day Q1 course or after). **Ongoing education and training n**<br>• Create and/or facilitate a community of practice. **Clinical facilitation**
	Physical	• Insufficient time within usual care to provide patient fall prevention interventions<br>• Insufficient ward staffing to provide patient fall prevention interventions<br>• Insufficient and appropriate resources to provide fall prevention (e.g. amount and state of mobility equipment, resources for CALD patients/families)	• Readily accessible resources on the ward to assist with fall prevention interventions (e.g. patient fall prevention education brochures, videos). **Clinical facilitation**<br>• Assistance navigating processes to source/maintain/ upgrade relevant resources. **Clinical facilitation**<br>• Exploring optimising ward staffing patterns. **Clinical facilitation**<br>• Assistance/ education regarding appropriate ways to increase patient and or visitors' involvement in fall prevention. **Ongoing education and training**
**Motivation**	Automatic (emotions, impulses)	• Belief that fall prevention is not part of professional role<br>• Belief that fall prevention is not an important aspect of patient care<br>• Belief that fall prevention not a priority within day-to-day workload<br>• Belief that falls cannot be prevented<br>• Incorrect assumptions about where, when, why falls occur	• Insights from Lived Experience Educator regarding consequences of sustaining a fall in hospital (in person session or viewing pre-recorded video). **Build a coalition**<br>• Feedback of patient fall prevention survey data to ward staff. **Clinical facilitation =**<br>• Ward based staff education by fall prevention experts and clinical facilitator (30 min sessions) and to staff on the go by PROTECT Fall Prevention ward leadership team (topics could include: fall prevention success stories, multidisciplinary roles in fall prevention, physical and psychological impact of a hospital fall). **Ongoing education and training**<br>• Discipline specific education sessions delivered by Fall Prevention Experts or clinical facilitator. **Ongoing education and training**

			• Display of ward specific falls data on the ward. **Quality improvement training**<br>• Discussion and feedback about fall prevention related performance at routine ward meetings. **Clinical facilitation**<br>• Fall prevention promotion display on ward. **Quality improvement training**<br>• Emphasis on celebration of fall prevention achievements (e.g. ward morning tea). **Clinical facilitation**<br>• Change management education to PROTECT Fall Prevention ward leadership team during or after 2-day Q1 course. **Ongoing education and training**<br>• Create and/or facilitate a community of practice. **Clinical facilitation**<br>• Provision and brainstorming of summary of local incident data. Clinical facilitation
	Reflexive (evaluations, plans)	Unaware of alignment of fall prevention interventions with:<br>• SLHD Fall Prevention and Management Strategy<br>• NSW Health CEC Older Persons Patient Safety Program<br>• ACSQHC Comprehensive Care Standard<br>• Accreditation<br>• Position descriptions	• SLHD Executive PROTECT Fall Prevention Program Steering Committee with multi-disciplinary and cross facility representation. **Build a coalition**<br>• Wards to engage an Executive sponsor/ champion for their involvement in the program (e.g. Facility Director of nursing) **Build a coalition**<br>• Attendance of PROTECT Fall Prevention ward leadership team at two-day quality improvement course. **Quality improvement training**<br>• Tailored ward-based staff education by Clinical facilitation. **Ongoing education and training**<br>• Discussion and feedback about fall prevention related performance at routine ward meetings. **Clinical facilitation**
