# Supplementary material for: Supported implementation of tailored hospital fall prevention interventions: a protocol for the PROTECT stepped wedge type I hybrid effectiveness-implementation trial
Source: BMJ Open. 2026 Mar 19;16(3):e111744. doi: 10.1136/bmjopen-2025-111744 (PMC13007140; doi:10.1136/bmjopen-2025-111744)
Supplement: online supplemental file 3 [file bmjopen-16-3-s003.pdf]

Supplementary material 3: Expanded table 2: Implementation strategies

<table>
<tr><td colspan="6">Core implementation strategy: Training (Quality improvement)
Branching implementation strategies: Capture and share local knowledge; conduct local consensus discussion; conduct cyclical small tests of change; develop and implement tools for quality monitoring; identify and prepare champions; model and simulate change; tailor strategies; audit and provide feedback
Action: Improvement Science training course is run by the Local Health District’s education department. Quality improvement is a system of iterative review and refinement aiming to produce positive changes to optimise local and organisational outcomes[1].
Training topics include: using diagnostic data to inform change ideas; assessing and implementing change ideas’ via Plan-Do-See-Act (PDSA) cycles; and assessing, spreading and sustaining improvement. The course will use mixed modes of training including presentations, workshops and interactive activities. The course will have 2 hours of additional hospital fall prevention evidence summary content added for the trial.</td></tr>
<tr><td>Actor</td><td>Target(s)</td><td>Temporality</td><td>Dose</td><td>Outcome affected</td><td>Justification</td></tr>
<tr><td>This training is a pre-existing course available to staff across the four participating hospitals run by clinicians who are experts in quality improvement and fall prevention. The primary trainer is an Educator working for the health district’s education department.</td><td>Minimum two clinicians from each participating ward who will be leading their team’s implementation of multicomponent fall prevention interventions.</td><td>Staff attend the course within the two-week period prior to the commencement of the intensive clinical facilitation support</td><td>Once off, two-day face-to-face course.</td><td>Adoption, reach, dose, sustainability</td><td>Research suggests quality improvement training is acceptable and feasible in supporting the implementation of multicomponent fall prevention interventions[2,3]. Face-to-face training is perceived to be more beneficial than online training[2].</td></tr>
<tr><td colspan="6">Core implementation strategy: Facilitation
Branching implementation strategies: Distribute educational materials; organise clinician implementation team meetings; promote adaptability; remind clinicians; use train-the-trainer strategies; create a learning collaborative
Action: A Clinical Facilitator provides ongoing training and support to clinical staff on improvement science methodology following their two-day training course. The facilitator continues guidance for teams (including and beyond the team members who attended the training course) about how an improvement science approach can support tailored fall prevention interventions in their clinical areas, accommodating their working environment. Training and education may include topics such as using available data to inform and evaluate change; practical understanding of</td></tr>
</table>

how to adapt ways of working to reflect their distinct ward/area setting, work culture, patient cohort and specific local challenges; implementing and managing change; and applying the theory of improvement science to falls prevention in a real-time clinical setting via PDSA cycles. The clinical facilitator can also provide the tailored ongoing fall prevention education and training described in the core implementation strategy below. Clinical facilitation is underpinned by theoretical models of behaviour change and involves an experienced clinician providing mentorship, guidance and support to clinical teams to support the uptake of positive changes in clinical practice[4].					
**Actor**	**Target(s)**	**Temporality**	**Dose**	**Outcome affected**	**Justification**
The Clinical Facilitator is a full-time, trial-funded position filled by a senior Nurse Specialist. Their workload is distributed across the four participating wards at a time.	Staff on participating wards implementing multi-component fall prevention interventions.	Clinical facilitation commences in week one of the participating wards' 17-week intensive implementation support period.	The clinical facilitator will meet face-to-face with the participating teams for at least 30minutes, once a week during the 17-week intensive implementation period and is available for access at other times on an as-needs basis. Teams then move to a minimum of 4 months of 30mins clinical facilitation per month (with additional support upon request).	Adoption, reach, dose, fidelity, sustainability	Clinical facilitation was successfully used to support multicomponent fall prevention intervention in a multi-site quality improvement project[3].<br>The team's prior research informing this study found clinical facilitation is acceptable and feasible in supporting the implementation of multicomponent fall prevention interventions[5].
**Core implementation strategy: Ongoing education and training**<br>**Branching implementation strategies:** Capture and share local knowledge; make training dynamic<br>**Action:** Tailored education about risks for and consequences of falling in hospital and training sessions on the practical delivery of ward-selected fall preventions strategies. Using adult learning techniques, staff education and training can increase staff engagement, skills, efficacy, confidence and motivation[6, 7].					
**Actor**	**Target(s)**	**Temporality**	**Dose**	**Outcome affected**	**Justification**
Education and training sessions are run by physiotherapy and	Staff on participating wards implementing multi-	Education and training sessions occur during the	The specific content, number and timing of sessions is informed by ward staff needs	Adoption, reach, dose, fidelity	Staff education and training is an integral component of the delivery

nursing members of the study team experienced in hospital fall prevention and by the Clinical Facilitator.	component fall prevention interventions.	intensive implementation phase	and preferences identified in the staff pre-implementation surveys, in discussion with ward staff and in testing their selected small local changes.		of effective fall prevention interventions in large trials in hospital settings[8, 9].<br>Staff education and training was identified as facilitator to multicomponent fall prevention in the team's prior research informing this study[2, 10].
**Core implementation strategy: Build a coalition**<br>**Action**: Internal and external stakeholders are engaged to optimise the implementation effort.<br>**Branching implementation strategies:** Identify and prepare champions; obtain and use patients/ consumers feedback; involve patients/consumers and family members; involve executive boards; promote network weaving; provide ongoing consultation; use advisory boards and workgroups; use an implementation advisor; work with educational institutions					
**Actor**	**Target(s)**	**Temporality**	**Dose**	**Outcome affected**	**Justification**
The research team intentionally map and engage stakeholders.	Internal and external stakeholders who will be involved, influence and/or impact the intervention and implementations strategies. Cohorts include but are not limited to academics, health executive and managers, clinicians, hospital staff, patients and families.	Commenced in months prior to study and in previous work informing the study. Continues throughout and after the study period.	The specific mode, number and timing of stakeholder interactions is dependent on stakeholder capacity and project needs.	Adoption, reach, dose, fidelity, sustainability.	The research team's relationships with broad stakeholder groups were crucial to the work informing this study[2, 10].<br>The implementation science field identifies a need for stakeholder engagement to optimise research and healthcare delivery[11].

REFERENCES

1. Puri I, Hollingshead, C, Tadi, P. . Quality Improvement. Treasure Island (FL) StatPEarls; 2023.
2. McLennan C, Sherrington C, Naganathan V, Tilden W, Richards B, McVeigh T, et al. Supported implementation of tailored multicomponent fall prevention interventions in hospital: a feasibility study. BMJ Open Qual. 2025;14(3).
3. Archer Y, Chasle B, Macpherson A, Wilson V. Advancing Quality and Safety Culture in Healthcare: Insights From the Evaluation of an Improvement Science Program for Nurses and Midwives. J Adv Nurs. 2025;81(6):3296-307.
4. Harvey G, Lynch E. Enabling Continuous Quality Improvement in Practice: The Role and Contribution of Facilitation. Front Public Health. 2017;5:27.
5. Curtis K, Fry M, Shaban RZ, Considine J. Translating research findings to clinical nursing practice. J Clin Nurs. 2017;26(5-6):862-72.
6. Shaw L, Kiegaldie D, Farlie MK. Education interventions for health professionals on falls prevention in health care settings: a 10-year scoping review. BMC Geriatr. 2020;20(1):460.
7. Fontaine G, Vinette B, Weight C, Maheu-Cadotte MA, Lavallee A, Deschenes MF, et al. Effects of implementation strategies on nursing practice and patient outcomes: a comprehensive systematic review and meta-analysis. Implement Sci. 2024;19(1):68.
8. Hill AM, McPhail SM, Waldron N, Etherton-Beer C, Ingram K, Flicker L, et al. Fall rates in hospital rehabilitation units after individualised patient and staff education programmes: a pragmatic, stepped-wedge, cluster-randomised controlled trial. Lancet. 2015;385(9987):2592-9.
9. Di Gennaro G, Chamitava L, Pertile P, Ambrosi E, Mosci D, Fila A, et al. A stepped-wedge randomised controlled trial to assess efficacy and cost-effectiveness of a care-bundle to prevent falls in older hospitalised patients. Age and Ageing. 2024;53(1).
10. McLennan C, Sherrington C, Tilden W, Jennings M, Richards B, Hill A-M, et al. Considerations across multiple stakeholder groups when implementing fall prevention programs in the acute hospital setting: a qualitative study. Age and Ageing. 2024;53(10).
11. Potthoff S, Finch T, Buhrmann L, Etzelmuller A, van Genugten CR, Girling M, et al. Towards an Implementation-STakeholder Engagement Model (I-STEM) for improving health and social care services. Health Expect. 2023;26(5):1997-2012.
