# Supplementary material for: Supported implementation of tailored hospital fall prevention interventions: a protocol for the PROTECT stepped wedge type I hybrid effectiveness-implementation trial
Source: BMJ Open. 2026 Mar 19;16(3):e111744. doi: 10.1136/bmjopen-2025-111744 (PMC13007140; doi:10.1136/bmjopen-2025-111744)
Supplement: online supplemental file 4 [file bmjopen-16-3-s004.pdf]

Supplementary material 4: Surveys and interview guides

PROTECT Fall Prevention Trial

Patient pre-implementation survey

Unfortunately, due to things such as illness, medication changes and the new surroundings patients can be at an increased risk of falling over while in hospital. This survey aims to improve our understanding of how we can best reduce the risk of patients falling over while in hospital. Thank you for taking the time to complete it**.**

☐ I have read the Participant Information Sheet
☐ I understand that in completing and returning the survey below, I am consenting to the survey.

1. **I am a:**
   - ☐ Patient
   - ☐ Family member
   - ☐ Carer

2. **I am:**
   - ☐ Male
   - ☐ Female
   - ☐ Non-binary
   - ☐ Prefer not to say

3. **My age is:**
   - ☐ 18-24
   - ☐ 25-34
   - ☐ 35-44
   - ☐ 45-54
   - ☐ 55-64
   - ☐ 65-74
   - ☐ 75-84
   - ☐ 85-94
   - ☐ 95 and over

4. **I am in ward**:
   [redacted]
   - ☐ Unsure

5. **How do you move about in hospital? :**
   - ☐ Immobile (in bed only)
   - ☐ Can move from bed to chair or walk with the assistance of 2 people (with or without a mobility aid)
   - ☐ Can move from bed to chair or walk with the assistance of 1 person or without a mobility aid)
   - ☐ Can move from bed to chair or walk using a mobility aid
   - ☐ Can move from bed to chair or walk on my own (that is, without the assistance of a person or mobility aid)
   - ☐ Other ____

6. **Please rate your agreement with the following statements on a scale of 1 to 5 with:**

   1 strongly disagree;
   2 disagree
   3 neither agree or disagree (neutral);
   4 agree
   5 strongly agree.

		1 (strongly disagree) to 5 (strongly agree)				
**1**	I could fall over while I am in hospital.	**1**	**2**	**3**	**4**	**5**
**2**	I understand how I can reduce my risk of falling over while in hospital.	**1**	**2**	**3**	**4**	**5**
**3**	I feel safe when I am moving about in hospital .	**1**	**2**	**3**	**4**	**5**
**4**	I feel confident asking for assistance from hospital staff.	**1**	**2**	**3**	**4**	**5**
**5**	Hospital staff encourage me to get out of bed.	**1**	**2**	**3**	**4**	**5**

7. **Is there anything that could be done to make you feel safer when moving about while in hospital?**

____

____

____

____

8. **What do you think could help reduce your risk of falling over while in hospital?** *(Select/tick any or all that apply)*

- ☐ Staff explaining to me why I am at risk of falling over in hospital
- ☐ Staff talking to me about ways to reduce my risk of falling over in hospital
- ☐ Staff talking to my visitors about ways to reduce my risk of falling over in hospital
- ☐ Watching a 5-minute video about ways to reduce the risk of falling over in hospital
- ☐ Feeling more confident to ask for help while in hospital
- ☐ Having a brochure with information about ways to reduce the risk of falling over in hospital
- ☐ Having access to more or different mobility equipment (e.g. walking frames)
- ☐ Other. Please specify ____

9. **Are there any other comments, concerns or suggestions that you have about preventing patients falling over while in hospital?**

____

____

____

____

END OF SURVEY.
MANY THANKS FOR YOUR PARTICIPATION.

PROTECT FALL PREVENTION PROGRAM

NSW GOVERNMENT | Health Sydney Local Health District

THE UNIVERSITY OF SYDNEY

PROTECT Fall Prevention Trial

Family pre-implementation survey

Unfortunately, due to things such as illness, medication changes and the new surroundings patients can be at an increased risk of falling over while in hospital. This survey aims to improve our understanding of how we can best reduce the risk of patients falling over while in hospital. Thank you for taking the time to complete it**.**

☐ I have read the Participant Information Sheet
☐ I understand that in completing and returning the survey below, I am consenting to the survey.

1. **I am a:**
   - ☐ Patient
   - ☐ Family member
   - ☐ Carer

2. **My family member is:**
   - ☐ Male
   - ☐ Female
   - ☐ Non-binary
   - ☐ Prefer not to say

3. **My family member's age is:**
   - ☐ 18-24
   - ☐ 25-34
   - ☐ 35-44
   - ☐ 45-54
   - ☐ 55-64
   - ☐ 65-74
   - ☐ 75-84
   - ☐ 85-94
   - ☐ 95 and over

4. **My family member is in ward**:
   [redacted]
   - ☐ Unsure

PROTECT
FALL PREVENTION PROGRAM

5. **How does your family member move about in hospital:**
   - ☐ Immobile
   - ☐ Can move from bed to chair or walk with the assistance of 2 people (with or without a mobility aid)
   - ☐ Can move from bed to chair or walk with the assistance of 1 person (with or without a mobility aid)
   - ☐ Can move from bed to chair or walk with a mobility aid
   - ☐ Can move from bed to chair or walk on their own (that is, without the assistance of a person or mobility aid)
   - ☐ Other ………………………………………………….

6. **Please rate your agreement with the following statements on a scale of 1 to 5 with:**

   1 strongly disagree;
   2 disagree
   3 neither agree or disagree (neutral);
   4 agree
   5 strongly agree.

		1 (strongly disagree) to 5 (strongly agree)				
**1**	My family member could fall over while in hospital.	**1**	**2**	**3**	**4**	**5**
**2**	I understand how I can help reduce the risk of my family member falling over while in hospital.	**1**	**2**	**3**	**4**	**5**
**3**	My family member is safe when moving about in hospital.	**1**	**2**	**3**	**4**	**5**
**4**	I feel confident asking for assistance from hospital staff.	**1**	**2**	**3**	**4**	**5**
**5**	My family member feels confident asking for assistance from hospital staff.	**1**	**2**	**3**	**4**	**5**
**6**	Hospital staff encourage my family member to get out of bed while.	**1**	**2**	**3**	**4**	**5**

7. **Is there anything that could be done to make your family member feel safer when moving about while in hospital?**

____

____

____

____

8. What do you think could help reduce the risk of your family member falling over while in hospital? *(Select/tick any or all that apply)*

- ☐ Staff explaining to me or my family member why they are at risk of falling over in hospital
- ☐ Staff talking to my family member about ways to reduce their risk of falling over in hospital
- ☐ Staff talking to me about ways to reduce my family member's risk of falling over in hospital
- ☐ Watching a 5minute video about ways to reduce risk of falling over in hospital
- ☐ Feeling more confident to ask for help while in hospital
- ☐ Having a patient and visitor brochure with information about ways to reduce risk of falling over in hospital
- ☐ Having access to more or different mobility equipment (e.g. walking frames)
- ☐ Other. Please specify ____

9. Are there any other comments, concerns or suggestions that you have about hospital falls prevention?

____

____

____

____

END OF SURVEY.
MANY THANKS FOR YOUR PARTICIPATION.

PROTECT Fall Prevention Trial

Patient post-implementation survey

Unfortunately, due to things such as illness, medication changes and the new surroundings patients can be at an increased risk of falling over while in hospital. This survey aims to improve our understanding of how we can best reduce the risk of patients falling over while in hospital. Thank you for taking the time to complete it.

☐ I have read the Participant Information Sheet
☐ I understand that in completing and returning the survey below, I am consenting to the survey.

1. **I am a:**
   - ☐ Patient
   - ☐ Family member
   - ☐ Carer

2. **I am:**
   - ☐ Male
   - ☐ Female
   - ☐ Non-binary
   - ☐ Prefer not to say

3. **My age is:**
   - ☐ 18-24
   - ☐ 25-34
   - ☐ 35-44
   - ☐ 45-54
   - ☐ 55-64
   - ☐ 65-74
   - ☐ 75-84
   - ☐ 85-94
   - ☐ 95 and over

4. **I am in ward:**

   [redacted]

   - ☐ Unsure

5. **How do you move about in hospital?**
   - ☐ Immobile (in bed only)
   - ☐ Can move from bed to chair or walk with help from 2 people (with or without a mobility aid)
   - ☐ Can move from bed to chair or walk with help from 1 person (with or without a mobility aid)
   - ☐ Can move from bed to chair or walk using a mobility aid
   - ☐ Can move from bed to chair or walk on my own (that is, without the assistance of a person or mobility aid)
   - ☐ Other --

6. **Please rate your agreement with the following statements on a scale of 1 to 5 with:**

   1. strongly disagree;
   2. disagree
   3. neither agree or disagree (neutral);
   4. agree
   5. strongly agree.

		1 (strongly disagree) to 5 (strongly agree)					
**1.**	I could fall over while I am in hospital.	**1**	**2**	**3**	**4**	**5**	**N/A**
**2.**	I understand how I can reduce my risk of falling over while in hospital.	**1**	**2**	**3**	**4**	**5**	**N/A**
**3.**	I feel safe when I am moving about in hospital.	**1**	**2**	**3**	**4**	**5**	**N/A**
**4.**	I feel confident asking for assistance from hospital staff.	**1**	**2**	**3**	**4**	**5**	**N/A**
**5.**	Hospital staff encourage me to get out of bed while in hospital.	**1**	**2**	**3**	**4**	**5**	**N/A**

7. **Have hospital staff have done anything to reduce your risk of falling over while in hospital?**
   - ☐ Yes
   - ☐ No

If yes:
7. A) How satisfied have you been with what has been done?

Please select/circle a response from 1 to 5 where 1= 'least satisfied' and 5 = 'most satisfied'

1	2	3	4	5

7. B) Can you briefly mention what hospital staff have done to reduce your risk of falling over while in hospital?

____

8. Is there anything that could be done to make you feel safer when moving about while in hospital?

9. What do you think could help reduce your risk of falling over while in hospital? *(Select/tick any or all that apply)*

- ☐ Staff explaining to me why I am at risk of falling over in hospital
- ☐ Staff talking to me about ways to reduce my risk of falling over in hospital
- ☐ Staff talking to my family about ways to reduce my risk of falling over in hospital
- ☐ Watching a 5minute video about ways to reduce the risk of falling over in hospital
- ☐ Feeling more confident to ask for help while in hospital
- ☐ Having a patient and visitor brochure with information about ways to reduce the risk of falling over in hospital
- ☐ Having access to more or different mobility equipment (e.g. walking frames)
- ☐ Other. Please specify ____

10. Are there any other comments, concerns or suggestions that you have about preventing patients falling over while in hospital?

END OF SURVEY.
MANY THANKS FOR YOUR PARTICIPATION.

PROTECT FALL PREVENTION PROGRAM | NSW GOVERNMENT Health Sydney Local Health District | THE UNIVERSITY OF SYDNEY

PROTECT Fall Prevention Trial

Family post-implementation survey

Unfortunately, due to things such as illness, medication changes and the new surroundings patients can be at an increased risk of falling over while in hospital. This survey aims to improve our understanding of how we can best reduce the risk of patients falling over while in hospital. Thank you for taking the time to complete it.

☐ I have read the Participant Information Sheet
☐ I understand that in completing and returning the survey below, I am consenting to the survey.

1. I am a
- ☐ Patient
- ☐ Family member
- ☐ Carer

2. My family member is:
- ☐ Male
- ☐ Female
- ☐ Non-binary
- ☐ Prefer not to say

3. My family member's age is:
- ☐ 18-24
- ☐ 25-34
- ☐ 35-44
- ☐ 45-54
- ☐ 55-64
- ☐ 65-74
- ☐ 75-84
- ☐ 85-94
- ☐ 95 and over

4. My family member is in ward:

[redacted]

- ☐ Unsure

5. **How does your family member move about in hospital?**
   - ☐ Immobile (in bed only)
   - ☐ Can move from bed to chair or walk with help from 2 people (with or without a mobility aid)
   - ☐ Can move from bed to chair or walk with help from 1 person (with or without a mobility aid)
   - ☐ Can move from bed to chair or walk using a mobility aid
   - ☐ Can move from bed to chair or walk on their own (that is without the assistance of a person or mobility aid)
   - ☐ Other ……………………………………………………

6. **Please rate your agreement with the following statements on a scale of 1 to 5 with:**

   1 strongly disagree;
   2 disagree
   3 neither agree or disagree (neutral);
   4 agree
   5 strongly agree.

		1 (strongly disagree) to 5 (strongly agree)					
**1**	My family member could fall over while in hospital.	**1**	**2**	**3**	**4**	**5**	**N/A**
**2**	I understand how I can help reduce the risk of my family member falling over while in hospital.	**1**	**2**	**3**	**4**	**5**	**N/A**
**3**	I feel my family member is safe when moving about on this hospital ward.	**1**	**2**	**3**	**4**	**5**	**N/A**
**4**	I feel confident asking for assistance from hospital staff.	**1**	**2**	**3**	**4**	**5**	**N/A**
**5**	My family member feels confident asking for assistance from hospital staff.	**1**	**2**	**3**	**4**	**5**	**N/A**
**6**	Hospital staff encourage my family member to get out of bed while in hospital.	**1**	**2**	**3**	**4**	**5**	**N/A**

7. **Have hospital staff done anything to reduce the risk of your family member falling over while in hospital?**
   - ☐ Yes
   - ☐ No

   If yes:
   7. A) How satisfied have you been with what has been done?

   Please select/circle a response from 1 to 5 where 1= 'least satisfied' and 5 = 'most satisfied'

1	2	3	4	5

7. B) Can you briefly mention what hospital staff have done to reduce your risk of falling over while in hospital?

____

____

____

____

8. Is there anything that could be done to make your family member feel safer when moving about while in hospital?

____

____

____

____

9. What do you think could help reduce the risk of your family member falling over while in hospital? *(Select/tick any or all that apply)*

- ☐ Staff explaining to me or my family member why they are at risk of falling over in hospital
- ☐ Staff talking to my family member about ways to reduce their risk of falling over in hospital
- ☐ Staff talking to me about ways to reduce my family member's risk of falling over in hospital
- ☐ Watching a 5minute video about ways to reduce risk of falling over in hospital
- ☐ Feeling more confident to ask for help while in hospital
- ☐ Receiving a patient and visitor brochure with information about ways to reduce risk of falling over in hospital
- ☐ Having access to more or different mobility equipment (e.g. walking frames)
- ☐ Other. Please specify ____

10. Are there any other comments, concerns or suggestions that you have about preventing patients falling over while in hospital?

____

____

____

____

END OF SURVEY.
MANY THANKS FOR YOUR PARTICIPATION.

PROTECT Fall Prevention Trial

Staff pre-implementation survey

I understand that in completing and returning the survey below, I am giving my implied consent as per the Participant Information Sheet I have read.

Please tick this box to confirm the above: ☐

1. **Please indicate your gender:**
   - ☐ Male
   - ☐ Female
   - ☐ Non-binary
   - ☐ Prefer not to say

2. **Please indicate your current professional area:**
   - ☐ Administrative Services
   - ☐ Allied Health
   - ☐ Environmental Services
   - ☐ Medicine
   - ☐ Nursing
   - ☐ Other

3. **Please select the ward where you currently work:**

   [redacted]

4. **Please indicate your years of experience in your current profession:**
   - ☐ 1-5 years
   - ☐ 6-10 years
   - ☐ 11-20 years
   - ☐ 21 years and over

PLEASE CONTINUE ON FOLLOWING PAGE

5. Please rate your agreement with the following statements on a scale of 1 to 5 with:

1. strongly disagree;
2. disagree
3. neither agree or disagree (neutral);
4. agree
5. strongly agree.

		1 (strongly disagree) to 5 (strongly agree)					
**1.**	It is possible to prevent patients from falling when in hospital.	**1**	**2**	**3**	**4**	**5**	**N/A**
**2.**	I understand my role in preventing patients from falling while in hospital.	**1**	**2**	**3**	**4**	**5**	**N/A**
**3.**	I feel confident undertaking fall prevention interventions on my ward.	**1**	**2**	**3**	**4**	**5**	**N/A**
**4.**	I am aware of the number, severity and location of recent falls on my ward.	**1**	**2**	**3**	**4**	**5**	**N/A**
**5.**	The fall prevention interventions provided on our ward are a good match for our **staff.**	**1**	**2**	**3**	**4**	**5**	**N/A**
**6.**	The fall prevention interventions provided on our ward are a good match for our **patients.**	**1**	**2**	**3**	**4**	**5**	**N/A**
**7.**	I am satisfied with the inpatient fall prevention interventions our ward implements.	**1**	**2**	**3**	**4**	**5**	**N/A**
**8.**	I am confident undertaking quality improvement projects.	**1**	**2**	**3**	**4**	**5**	**N/A**
**9.**	I am confident in assessing patients' falls risks.	**1**	**2**	**3**	**4**	**5**	**N/A**
**10.**	I am confident in selecting fall prevention strategies based on identified patient falls risks.	**1**	**2**	**3**	**4**	**5**	**N/A**
**11.**	The FRAMP is useful in reducing a patient's falls risk.	**1**	**2**	**3**	**4**	**5**	**N/A**
**12.**	The fall prevention interventions I implement on my ward are informed by the SLHD Fall Risk and Management Strategy.	**1**	**2**	**3**	**4**	**5**	**N/A**
**13.**	I have the support I need from other personnel to care for patients.	**1**	**2**	**3**	**4**	**5**	**N/A**
**14.**	The physicians, nurses and allied health professionals here work together as a well-coordinated team.	**1**	**2**	**3**	**4**	**5**	**N/A**
**15.**	I know the proper channels to direct questions regarding patient fall prevention.	**1**	**2**	**3**	**4**	**5**	**N/A**
**16.**	The culture in this clinical area makes it easy to learn from the errors of others.	**1**	**2**	**3**	**4**	**5**	**N/A**

**17.**	This is a good place to work.	**1**	**2**	**3**	**4**	**5**	**N/A**
**18.**	I feel happy at work.	**1**	**2**	**3**	**4**	**5**	**N/A**
**19.**	I feel worthwhile at work.	**1**	**2**	**3**	**4**	**5**	**N/A**
**20.**	My work is satisfying to me.	**1**	**2**	**3**	**4**	**5**	**N/A**
**21.**	I feel in control when dealing with difficult problems at work.	**1**	**2**	**3**	**4**	**5**	**N/A**
**22.**	I'm contributing professionally (e.g. patient care, teaching, research, leadership) in the ways I value most.	**1**	**2**	**3**	**4**	**5**	**N/A**
**23.**	I feel exhausted at work.	**1**	**2**	**3**	**4**	**5**	**N/A**
**24.**	My job contributes to me feeling less empathetic with my colleagues.	**1**	**2**	**3**	**4**	**5**	**N/A**
**25.**	Communication breakdowns that lead to delays in delivery of care are common.	**1**	**2**	**3**	**4**	**5**	**N/A**

6. **What are the main reasons it can be difficult to prevent patient falls on your ward?**

____

____

____

7. **What are ways that may help you or your ward provide optimal patient fall prevention interventions?**

____

____

____

8. **In a typical week, how frequently do you implement effective interventions to reduce patients falls risk?** *(please tick box your answer)*

Frequently (>75% of the time)	Often (50-74% of the time)	Sometimes (25-49% of the time)	Rarely (1-24% of the time)	Never (0%)

PLEASE CONTINUE ON FOLLOWING PAGE

9. **What do you think helps, or would help, you to implement effective fall prevention interventions on your ward?** *(Select/tick any or all that apply)*

- ☐ Education sessions about effective fall prevention care/ activities
- ☐ Regular reminders about fall prevention care/ activities
- ☐ Feedback about recent falls on my ward
- ☐ Feeling confident about carrying out fall prevention care/ activities
- ☐ Educational resources available on the ward that assist with falls prevention care/ activities
- ☐ Having access to someone to ask questions about fall prevention
- ☐ Group reflection on recent falls on the ward
- ☐ Scenario-based learning
- ☐ Other. Please specify ____
  ____

10. **Are there any other comments, concerns or suggestions that you have about hospital fall prevention?**

____

____

____

END OF SURVEY.
MANY THANKS FOR YOUR PARTICIPATION.

PROTECT Fall Prevention Trial

Staff post-implementation survey

I understand that in completing and returning the survey below, I am giving my implied consent as per the Participant Information Sheet I have read.

Please tick this box to confirm the above: ☐

1. **Please indicate your gender:**
   - ☐ Male
   - ☐ Female
   - ☐ Non-binary
   - ☐ Prefer not to say

2. **Please indicate your current professional area:**
   - ☐ Administrative Services
   - ☐ Allied Health
   - ☐ Environmental Services
   - ☐ Medicine
   - ☐ Nursing
   - ☐ Other

3. **Please indicate your years of experience in your current profession:**
   - ☐ 1-5 years
   - ☐ 6-10 years
   - ☐ 11-20 years
   - ☐ 21 years and over

4. **Are you a member of the PROTECT team on your ward?**
   - ☐ Yes
   - ☐ No
   - ☐ Prefer not to say

5. Please rate your agreement with the following statements on a scale of 1 to 5 with:

1. being strongly disagree;
2. disagree;
3. neither agree or disagree (neutral);
4. agree;
5. strongly agree.

		1 (strongly disagree) to 5 (strongly agree)					
1.	It is possible to prevent patients from falling when in hospital.	**1**	**2**	**3**	**4**	**5**	**N/A**
2.	I understand my role in preventing patients from falling while in hospital.	**1**	**2**	**3**	**4**	**5**	**N/A**
3.	I feel confident undertaking fall prevention interventions on my ward.	**1**	**2**	**3**	**4**	**5**	**N/A**
4.	I am aware of the number, severity and location of recent falls on my ward.	**1**	**2**	**3**	**4**	**5**	**N/A**
5.	The fall prevention interventions provided on our ward are a good match for our **staff.**	**1**	**2**	**3**	**4**	**5**	**N/A**
6.	The fall prevention interventions provided on our ward are a good match for our **patients.**	**1**	**2**	**3**	**4**	**5**	**N/A**
7.	I am satisfied with the strategies our ward implements to prevent patient falls	**1**	**2**	**3**	**4**	**5**	**N/A**
8.	I am confident undertaking quality improvement projects.	**1**	**2**	**3**	**4**	**5**	**N/A**
9.	I am confident in assessing patients' falls risks.	**1**	**2**	**3**	**4**	**5**	**N/A**
10.	I am confident in selecting fall prevention strategies based on identified patient falls risks.	**1**	**2**	**3**	**4**	**5**	**N/A**
11.	The e-FRAMP is useful in reducing a patient's falls risk.	**1**	**2**	**3**	**4**	**5**	**N/A**
12.	The fall prevention interventions I implement on my ward are informed by the SLHD Fall Risk and Management Strategy.	**1**	**2**	**3**	**4**	**5**	**N/A**
13.	I have the support I need from other personnel to care for patients.	**1**	**2**	**3**	**4**	**5**	**N/A**
14.	The physicians, nurses and allied health professionals here work together as a well-coordinated team.	**1**	**2**	**3**	**4**	**5**	**N/A**
15.	I know the proper channels to direct questions regarding patient fall prevention.	**1**	**2**	**3**	**4**	**5**	**N/A**
16.	The culture in this clinical area makes it easy to learn from the errors of others.	**1**	**2**	**3**	**4**	**5**	**N/A**
17.	This is a good place to work.	**1**	**2**	**3**	**4**	**5**	**N/A**
18.	I feel happy at work.	**1**	**2**	**3**	**4**	**5**	**N/A**
19.	I feel worthwhile at work.	**1**	**2**	**3**	**4**	**5**	**N/A**

20.	My work is satisfying to me.	**1**	**2**	**3**	**4**	**5**	**N/A**
21.	I feel in control when dealing with difficult problems at work.	**1**	**2**	**3**	**4**	**5**	**N/A**
22.	I'm contributing professionally (e.g. patient care, teaching, research, leadership) in the ways I value most.	**1**	**2**	**3**	**4**	**5**	**N/A**
23.	I feel exhausted at work.	**1**	**2**	**3**	**4**	**5**	**N/A**
24.	My job contributes to me feeling less empathetic with my colleagues.	**1**	**2**	**3**	**4**	**5**	**N/A**
25.	Communication breakdowns that lead to delays in delivery of care are common.	**1**	**2**	**3**	**4**	**5**	**N/A**

6. In a typical week, how frequently do you implement effective interventions to reduce patients falls risk?

Frequently (>75% of the time)	Often (50-74% of the time)	Sometimes (25-49% of the time)	Rarely (1-24% of the time)	Never (0%)

7. Please indicate the ward where you currently work:

[redacted]

8. Are you aware of the recent PROTECT falls prevention program on your ward?

- ☐ Yes
- ☐ No

If yes:

Some of the strategies your ward has been implementing with the PROTECT Fall prevention program include:

[redacted]

A) Have you been delivering/ participating in the strategies listed above when appropriate/ indicated?

- ☐ Yes
- ☐ No
- ☐ Sometimes

B) Do you think the strategies above have been implemented as planned?

- ☐ Yes
- ☐ No
- ☐ Partly
- ☐ Unsure

C) How satisfied have you been with the support provided to implement the strategies above?

Please select/circle a response from 1 to 5
where 1= 'least satisfied' and 5 = 'most satisfied'

1	2	3	4	5

D) Has the PROTECT fall prevention program had a positive change on clinical practice on your ward?

- ☐ Yes
- ☐ No
- ☐ Partly

E) How would you rate the impact of the PROTECT Fall prevention program on positive changes in your clinical practice?

Please select/circle a response from 1 to 5
where 1= 'no impact at all' and 5 = 'major impact on my clinical practice'

1	2	3	4	5

F) Do you think the positive changes will continue once the PROTECT program finishes?

- ☐ Yes
- ☐ No
- ☐ Partly

Why/ why not?

____

____

____

____

G) How likely are you to continue any positive changes in practice that have come about from the PROTECT fall prevention program?

Please select/circle a response from 1 to 5
where 1= 'not likely at all' and 5 = 'extremely likely'

1	2	3	4	5

H) Do you intend to use the quality improvement methods used in PROTECT to assist changes in practice on your ward in the future?

- ☐ Yes
- ☐ No
- ☐ Unsure

I) Since the start of the PROTECT fall prevention program on your ward, have you noticed patients are mobilising:

- ☐ More than prior to the program
- ☐ Less than prior to the program
- ☐ About the same

J) What worked well for you and/or your ward during the PROTECT fall prevention program?

____

____

____

____

K) **What did not work well for you and/or your ward during the PROTECT fall prevention program?**

____

____

____

____

9. **Are there any other comments, concerns or suggestions that you have about the PROTECT fall prevention program or preventing patient falls in hospitals?**

____

____

____

____

END OF SURVEY.
MANY THANKS FOR YOUR PARTICIPATION.

PROTECT FALL PREVENTION TRIAL

STAFF Interview/ focus group guide

Thank you for agreeing to participate in this interview/ focus group. My name is ________________________ and my role is_________________ . I am conducting interviews/ focus groups to explore the impact of the recent PROTECT Fall Prevention Program on the ward you work on.

I will ask questions during the discussion that you can answer however you would like. All of the information we collect in this interview will be keep confidential. The interview should take about 30 minutes to complete OR the focus group should take about 30-60minutes to complete. As described in the Participant Information Sheet and the consent form, I will be audio-recording the interview so that I can concentrate on your responses and refer back to them during analysis. Can I confirm again that this is okay with you?

If there are any questions you wish to skip or come back to at a later time, or if you need to take a break at any time, please let me know. We can also cease the interview at any time if you become uncomfortable.

Do you have any questions before we begin?

PART ONE: PROTECT Team

1. A) Can you tell me about the role you played in the PROTECT Fall Prevention Program on your ward XXX?

B) Can you tell me about the rest of the team that led the PROTECT program on your ward? *(Can you tell me about the roles other people on your ward played in the PROTECT program)?*

C) What worked well about or for your team? *What worked well about the PROTECT leadership team?*

E) What did not work well for or about your team? *What did not work well about the PROTECT leadership team?*

PART TWO: Implementation support

The next questions relate to the implementation (or support) strategies that were used to support your ward to implement selected fall prevention interventions (or change ideas). The implementation strategies included:

- 2-day Improvement Science training course
- 4 months of weekly support from Sally (PROTECT Clinical Facilitator)
- Provision of a ward falls profile that summarised data from falls on your ward over the last 2 years and some patient and staff survey feedback.
- QIDS platform to monitor progress with change ideas

2, 3, 4) We'll go through these implementation strategies one by one now and can you share what you found useful about them. And then anything you didn't like, or that didn't work about them. Additionally if there is anything you'd change about them for the future.

5) Do you have any reflections on how these strategies supported you as a bundle?

OR:

2.Thinking about these implementation/ support strategies, what thoughts do you have on their usefulness?

Prompts: Change in confidence to implement fall prevention?

Change in motivation to implement fall prevention?

Change in knowledge/ skills to implement fall prevention?

Change in ease/ ability to implement fall prevention?

3. A) Were there aspects of these implementation/ support strategies (that is improvement science training days, weekly meetings etc.) **that did not work well?**

Are there things you'd change about the support, things you didn't like about it?

B) Did you make any changes because of these issues? Would you make any future changes?

PART THREE: Change ideas

The next questions relate to the change ideas that have been rolled out on your ward during the PROTECT program.

4. **Can you describe the initiatives or change ideas your ward has implemented during the PROTECT program?**

5. **A) What impact have these changes had?** *Prompts: impact on staff? Impact on patient care?*

   B) Have you noticed any particular impact on patient care? Have patients been receptive to any changes in care that have come about due to the PROTECT program?

   C) **Can you comment on the impact of the PROTECT program on patients' mobility on your ward?** *Have you noticed if patients have been mobilising more, less or the same? Reasons for this?*

6. **A) What have been the challenges, if any, to implementing these change ideas on your ward?**

B) Have you been able to overcome these challenges? How?

7. **Do you feel interventions to reduce patient falls on your ward can be delivered routinely over time? Why/why not?** *How can sustained implementation be facilitated (on your ward and across different settings)? Are there things that can be done to help embed changes long term?*

PART FOUR: Overall

We've talked about what went well and what didn't in specific parts of PROTECT. Our last questions now just ask you to think about the program overall and see if there are any final reflections.

8. **Thinking about the overall PROTECT fall prevention program:**

   A) How successful do you think the program has been on your ward?
   What outcomes/ consequences has the program had for your ward.
   - ➔ *If they don't mention success in relation to patients falling, probe about this.*
   - ➔ *Can also probe about any unexpected outcomes from the program.*

   B) What contributed to this success (or lack of success)?
   In addition to things you have mentioned in the interview already, is there anything else that contributed to this success (or lack of success)?

   C) **What would you change about the PROTECT fall prevention program for future wards?** *What didn't work well, how could it work better?*

9. **That brings us to the end of the interview unless there is there anything else you would like to add?**

Thank you for your time and valuable insights.

PATIENT/ FAMILY Interview/ focus group guide

Thank you for agreeing to participate in this interview/ focus group. My name is ________________________ and my role is_________________ .I am conducting interviews to explore the impact of a program we have been undertaking with staff on this ward you are (or the person you are visiting is) admitted on, on to implement tailored strategies to reduce patients risk of having a fall while they are in hospital.

I will ask questions during the discussion that you can answer however you would like. All of the information we collect in this interview will be keep confidential. The interview should take about 30 minutes to complete OR the focus group should take about 30-60minutes to complete. As described in the Participant Information Sheet and the consent form, I will be audio-recording the interview so that I can concentrate on your responses and refer back to them during analysis. Can I confirm again that this is okay with you?

If there are any questions you wish to skip or come back to at a later time, or if you need to take a break at any time, please let me know. We can also cease the interview at any time if you become uncomfortable.

Do you have any questions before we begin?

1. Prior to coming into hospital, what were your thoughts about falls in hospital. Has this changed since you/ the person you are visiting have been admitted? Why/ Why not?

2. Have you ever worried about (t*he person you are visiting*) falling over while in hospital? Why/ why not?
   Have you *(Has the person you are visiting)* ever fallen over while in hospital? If yes, can you tell me about this? What has been the long term impact of this?

3. Have you noticed anything staff have been doing to try to reduce your *(or the person you are visiting)* risk of falling while in hospital? If no, can prompt if noticed anything to help keep them safe when moving around?
   If yes:
   Can you tell me about what they did and how it made you*/ the person you were visiting* feel?
   Prompts: Usefulness of what was done, change in knowledge/ skills, change in ease/ ability to do things, change in motivation to prevent falls
   What have been the challenges associated with the strategies you have mentioned? Have these been able to be overcome? How?

4. What are some ways, if any, that staff could have made you (or other patients/ the person you are visiting) feel safer in regard to falling in hospital (or can frame as feel safer when moving around?)
