# Supplementary material for: Supported implementation of tailored hospital fall prevention interventions: a protocol for the PROTECT stepped wedge type I hybrid effectiveness-implementation trial
Source: BMJ Open. 2026 Mar 19;16(3):e111744. doi: 10.1136/bmjopen-2025-111744 (PMC13007140; doi:10.1136/bmjopen-2025-111744)
Supplement: Supplementary file 5 [file bmjopen-2025-111744.reviewer_comments.pdf]

PEER REVIEW HISTORY

BMJ Open publishes all reviews undertaken for accepted manuscripts. Reviewers are asked to complete a checklist review form and are provided with free text boxes to elaborate on their assessment. These free text comments are reproduced below.

ARTICLE DETAILS

Title (Provisional)

Supported implementation of tailored hospital fall prevention interventions: a protocol for the PROTECT stepped wedge type I hybrid effectiveness-implementation trial

Authors

McLennan, Charlotte; Hassett, Leanne; Tilden, Wendy; Naganathan, Vasi; Haynes, Abby; Jennings, Matthew; Ni Chroinin, Danielle; Richards, Bethan; Hallahan, Andrew; Biswas, Raaj Kishore; Kwok , Wing; McVeigh, Tamsin; Heppleston , Erica; Jackson, Debra; Nayak , Veethika; Delaney , Sally; Howard, Kirsten; Pinheiro , Marina; Macpherson, Amanda; Rayner, Jenny; Hill, Anne-Marie; Haines, Terry; Sherrington, Catherine

---

VERSION 1 - REVIEW

**Reviewer**	**1**
**Name**	**Wingood, Mariana**
**Affiliation**	**Wake Forest School of Medicine, Department of Implementation Science**
**Date**	**25-Nov-2025**
**COI**	**None**

Introduction

-General recommend re-writing the introduction in a more direct manner, using less filler words. For example: “They can lead to significant physical injuries, especially in older people, and sometimes death.” The sentence starts with “they can lead” this is very similar to sometimes, thus, the “sometimes” is not necessary. The authors can state “They can lead to significant physical injuries and death, especially among older people.” Another example is the second sentence; by placing a clause in the middle of the sentence it increases cognitive load. Thus, it is recommended to rewrite the sentence as “Even without injuries, hospital falls can reduce confidence and activity levels, increase length…..”

-In the third paragraph the authors start talking about the importance of targeted implementation strategies and then in the next sentence they discuss specific pre-set strategies that do not appear to be targeted. When examining the references for this section

it appears that there is a mix of references that do not fully support this statement. For example, the CFIR is sited which would mean that contextual determinants are being considered, yet this is not visible or mentioned in this paragraph.

Methods

-It is recommended to expand on the pragmatic nature of this design. There are many benefits of utilizing a pragmatic design for this study, including external validity and increase likelihood of scale-up. Feasibility may be a reason for selecting this design, but it should not be the primary reason for selecting this design.

-Similarly, for grouping the clusters. In addition to feasibility, there are other benefits for utilizing cluster randomization, such as decreased risk of contamination. I know feasibility is an easy reason for deciding to go one way or another when it comes to selecting study designs/methods, but I highly recommend that the authors and research team try to think beyond feasibility.

-It is interesting to see the wash-in occur after the implementation strategies have occurred. Could you provide additional reason for this. It seems that data collection and wash-in would occur prior to any implementation strategies occurring. Based on your figure 1, it does appear that the data collection and wash-in is occurring prior to the implementation support.

-Could you define what allied health professionals you are referring to? There is currently a trend to not using “allied health professionals” as it implies that these professionals are secondary or supporting to others and many traditional “allied health professionals” such as physical therapists, occupational therapists, and dietitians see themselves as independent practitioners and not “allied” to medicine. It also indicates a lack of inclusivity as it does not clearly state who it is referring to. Thus, many organizations and individuals prefer the term “interprofessional healthcare team” or “health and rehabilitation professionals” or just listing them all. Personally, for a protocol I would recommend listing all the professionals involved as it increases transparency and replicability.

-Please provide more information about the co-design. For example, what framework is being used, what steps will be followed, what techniques/methods (i.e., workshops, focus groups, design sprints, iterative prototyping), what is the decision-making process, etc.

-How were these implementation strategies selected? Are they co-designed? Do the key partners get an input about what implementation strategies have worked in their wards in the past and what haven’t and what strategies they prefer/don’t prefer? When describing the implementation strategies, please use the recommended frameworks/model for describing implementation strategies. These could be the Proctor’s specification guidelines that include the following key elements: name of the strategy, actor, action, target, temporality, dose, and justification.

-Related to implementation data, any data collected related to identifying which strategies work and which do not? How will you know how to fine-tune the implementation strategies?

-I am excited to see cost-effectiveness being included in this study and protocol as it is an essential aspect of both sustainability and scale-up. However, I also feel like it was just thrown in there/it appears to come out of nowhere on page 17. Thus, I recommend that either this piece is woven in more throughout the manuscript or removed from the manuscript. For example, there is no information about cost in the introduction. If cost-effectiveness is part of the study, then it is recommended that it is discussed from the start. The methods also need to expanded upon. For example, whose perspective is this coming from (e.g., societal, healthcare system, payer, or patient). Is there a comparator? What costs are included? What are the outcome measures? Are you using CHEERS guidelines?

VERSION 1 - AUTHOR RESPONSE

Peer review comment	Response
General recommend re-writing the introduction in a more direct manner, using less filler words. For example: "They can lead to significant physical injuries, especially in older people, and sometimes death." The sentence starts with "they can lead" this is very similar to sometimes, thus, the "sometimes" is not necessary. The authors can state "They can lead to significant physical injuries and death, especially among older people." Another example is the second sentence; by placing a clause in the middle of the sentence it increases cognitive load. Thus, it is recommended to rewrite the sentence as "Even without injuries, hospital falls can reduce confidence and activity levels, increase length....."	The introduction has been re-written in a more direct manner. It now reads:<br>**Pages 4&5**<br>"*Falls in hospitals remain a common and costly problem for health systems, health staff and patients and their families (1, 2). They can lead to significant physical injuries and death, especially in older people(3, 4). Even without injuries, hospital falls can reduce confidence, activity levels and independence, increase length of stay, and alter discharge destinations (5). No single approach or intervention has proved a solution for this complex problem across broad acute and subacute hospital patient populations (6-8). This is reflective of the diversity and complexity of patients and contexts in which falls occur.*<br>*The recent World Falls Guidelines (4) highlight the need for hospital fall prevention strategies that are tailored to specific environments and personalised for individual patients. Tailored multicomponent fall prevention interventions, especially those involving patient education show a promising impact on hospital falls (9, 10). However, individualised patient education is time consuming and unlikely to be cost-effective on acute wards with rapid through-put and a lower total proportion of people who fall in hospital (11). Large trials of patient education*

	interventions have also had inconsistent results in patients with cognitive impairment (9, 12, 13). Among hospital inpatients 65 and over, 29-38% are estimated to have cognitive impairment (14). One study across a large Australian metropolitan hospital found 43% of inpatients over 65 demonstrated cognitive impairment (15). The prevalence of inpatients with cognitive impairment is likely to grow with the growing proportion of hospital admissions by older people (16).<br>*The implementation of tailored multicomponent fall prevention interventions into routine practice can be challenging and variable (17, 18). Targeted implementation strategies that are rigorously designed and address barriers to implementation are required to support this process (2, 19, 20). The research team's prior work explored barriers and facilitators to delivery of multicomponent hospital fall prevention interventions in four local hospital wards, similar in context to the wards planned for this trial (18, 21). The barriers and facilitators were mapped to the COM-B model of behaviour change to identify suitable implementation strategies targeting ward level capabilities, opportunities and motivation (22). The strategies were then tested in a feasibility study in those wards and further refined based on the results. Subsequent mapping (included in supplementary material 1) identified four core implementation strategies that when tailored in collaboration with local ward staff to their local context may support effectiveness of the tailored multicomponent fall interventions in this trial. These include quality improvement training; clinical facilitation; ongoing training and education; and building a coalition. These implementation strategies are intended to build workforce capacity for developing and sustaining change (23).*<br>*Economic evaluations are crucial to informing the cost-effectiveness, or value for money, of hospital fall prevention interventions and implementation strategies, thus providing critical evidence for decision-making in policy and practice, especially at scale (24). Guidelines for economic*

	evaluation of fall prevention strategies have been available since 2011(25). Despite this, few trials of interventions to reduce falls in hospital settings incorporate economic evaluations (7).<br>*This paper describes the protocol for a stepped-wedge trial that will evaluate whether supported implementation (quality improvement training, clinical facilitation, ongoing education and training and building a coalition) of tailored ward-based multicomponent fall prevention interventions reduces patient falls in hospitals. The trial will also evaluate the impact of supported implementation of tailored ward-based multicomponent fall prevention interventions on a range of secondary effectiveness outcomes, and implementation outcomes and determinants. The cost-effectiveness of the program from a hospital perspective will also be evaluated."*
In the third paragraph the authors start talking about the importance of targeted implementation strategies and then in the next sentence they discuss specific pre-set strategies that do not appear to be targeted. When examining the references for this section it appears that there is a mix of references that do not fully support this statement. For example, the CFIR is sited which would mean that contextual determinants are being considered, yet this is not visible or mentioned in this paragraph.	We agree that the introduction of the implementation strategies and their justification was insufficient. We have changed the introduction text to explain how these implementation strategies were identified in collaboration with local ward staff:<br>**Page 5**<br>"*The implementation of tailored multicomponent fall prevention interventions into routine practice can be challenging and variable (17, 18). Targeted implementation strategies that are rigorously designed and address barriers to implementation are required to support this process (2, 19, 20). The research team's prior work explored barriers and facilitators to delivery of multicomponent hospital fall prevention interventions in four local hospital wards, similar in context to the wards planned for this trial (18, 21). The barriers and facilitators were mapped to the COM-B model of behaviour change to identify suitable implementation strategies targeting ward level capabilities, opportunities and motivation (22). The strategies were then tested in a feasibility study in those wards and further refined based on the results.*

	Subsequent mapping (included in supplementary material 1) identified four core implementation strategies that when tailored in collaboration with local ward staff to their local context may support effectiveness of the tailored multicomponent fall interventions in this trial. These include quality improvement training; clinical facilitation; ongoing training and education; and building a coalition. These implementation strategies are intended to build workforce capacity for developing and sustaining change (23)."<br><br>Thank you for pointing out the error with our references. We have reviewed these and now removed reference to the CFIR as while contextual determinants were considered we do not have formal modelling against the CFIR to include with the manuscript. The only framework we now reference is the COM-B model of behaviour change. We have kept references supporting the importance of targeted implementation strategies in healthcare and fall prevention.<br><br>We have added the work we did mapping the barriers to the implementation strategies as **Supplement 1** in the supplementary material.<br><br>We have also added an additional implementation strategy, (building a coalition) that in meetings while the protocol was under review, the research team agreed was being conducted but not adequately reported. We have removed learning collaboratives as an overarching implementation strategy as the research team agreed this better fit as a potential branching strategy under facilitation.<br><br>We have deleted some text in the introduction that is now presented in Table 3: Implementation strategies. This provides thorough specification and reporting of the implementation strategies as per Proctor et al.'s recommendations.<br><br>We have also added to the methods text to better explain the implementation strategies.

	In addition to the updated Table 3, the text has been updated:<br>**Page 11**<br>*"The multi-faceted implementation approach is delivered to ward staff/clinicians to support them to implement their selected tailored fall prevention interventions into their local ward. The approach involves overarching implementation strategies of quality improvement training, clinical facilitation, ongoing staff education and training, and the enduring building and maintenance of a coalition. Details of the strategies and how they may be further developed or tailored are presented in table 3 following Proctor's et al.'s recommendations for reporting implementation strategies (52). Each overarching strategy may branch into or incorporate additional strategies defined as per the ERIC compilation of implementation strategies (53). Additional implementation strategies may be created to meet individual ward needs based on their pre implementation needs profile and selected tailored interventions."*
It is recommended to expand on the pragmatic nature of this design. There are many benefits of utilizing a pragmatic design for this study, including external validity and increase likelihood of scale-up. Feasibility may be a reason for selecting this design, but it should not be the primary reason for selecting this design.	Thank you for this helpful recommendation. We have expanded on the reasoning for the pragmatic design. It now includes the following text and additional references:<br>**Pages 5&6**<br>*"This study design was chosen as it involves recruitment and intervention implementation at a ward level which, in a context of an intervention that has ward-level impacts, can lead to less bias in the results (28, 30). The pragmatic design allows enrolment of diverse patient and staff populations, and embeds the research within everyday clinical workflows, increasing the external validity, generalisability and scalability of the findings (31, 32). The design also allows for the implementation support to be delivered at staggered times, making delivery more feasible (33)."*<br>*31. Casey JD, Beskow LM, Brown J, Brown SM, Gayat E, Ng Gong M, et al. Use of pragmatic and explanatory trial designs in acute care research: lessons from COVID-19. Lancet Respir Med. 2022;10(7):700-14.*

	32. Fernandes A, Bartlett ES, Nichol G. Pragmatic vs Explanatory Trials. JAMA Cardiol. 2020;5(4):487-8.<br>*33. Weijer C, Taljaard M. Ethical Challenges Associated with Pragmatic and Cluster RCTs. N Engl J Med. 2024;391(11):969-71.*
Similarly, for grouping the clusters. In addition to feasibility, there are other benefits for utilizing cluster randomization, such as decreased risk of contamination. I know feasibility is an easy reason for deciding to go one way or another when it comes to selecting study designs/methods, but I highly recommend that the authors and research team try to think beyond feasibility.	We have adjusted the wording in the methods to provide more compelling justification for using cluster randomisation in this trial. The methods now include the following detail and references:<br>**Page 6**<br>*"Wards will be grouped into clusters of four based on geographic proximity. <u>This clustering approach will ensure feasibility of intervention delivery while also minimising risk of contamination bias and promoting coherence with ward and organisational processes (36, 37)"</u>*<br>36. Hemming K, Taljaard M. Key considerations for designing, conducting and analysing a cluster randomized trial. Int J Epidemiol. 2023;52(5):1648-58.<br>37. Hayes R, Moulton, LH. Cluster Randomised Trials, Second Edition: Chapman and Hall/CRC; 2017.
It is interesting to see the wash-in occur after the implementation strategies have occurred. Could you provide additional reason for this. It seems that data collection and wash-in would occur prior to any implementation strategies occurring. Based on your figure 1, it does appear that the data collection and wash-in is occurring prior to the implementation support.	The wash-in periods will occur prior to the intensive implementation support. There is the commencement of some components of the implementation support during the wash-in period. These include data collection for the ward falls profiles and the improvement science training. We have provided greater clarification and justification of the timing of the implementation support and wash in periods in the methods text:<br>**Page 6**<br>"*The majority of the implementation strategies will be delivered to ward staff during the 4-month implementation support periods. There will be a wash-in period for one month prior to the formal implementation period of each cluster to allow for collation of data to inform ward fall profiles and for some initial tailoring of implementation and intervention strategies. <u>Wards will commence improvement science training in this window to ensure they have</u>*

	baseline knowledge to optimise support from the clinical facilitator at the start of the intensive implementation support period. Primary outcome data will not be collected during the wash-in period as this period is not clearly control phase or clearly intervention phase data."<br>This aligns with the details of the wash-in period provided in Table 1: Stepped-wedge trial design and Figure 1: Study flowchart.
Could you define what allied health professionals you are referring to? There is currently a trend to not using "allied health professionals" as it implies that these professionals are secondary or supporting to others and many traditional "allied health professionals" such as physical therapists, occupational therapists, and dietitians see themselves as independent practitioners and not "allied" to medicine. It also indicates a lack of inclusivity as it does not clearly state who it is referring to. Thus, many organizations and individuals prefer the term "interprofessional healthcare team" or "health and rehabilitation professionals" or just listing them all. Personally, for a protocol I would recommend listing all the professionals involved as it increases transparency and replicability.	The allied health professionals we are referring to for this trial include physiotherapy, occupational therapy, dietetics, speech pathology, social work, pharmacy, podiatry, orthotics, diversional therapy. We have altered the relevant sections of the methods to specify this. This includes:<br>**Page 7**<br>*"Staff from nursing, medical, physiotherapy, occupational therapy, dietetics, speech pathology, social work, pharmacy, podiatry, orthotics, diversional therapy, administration and environmental services disciplines working on the participating wards, and inpatients and their family members on the participating wards, will be invited to complete pre- and post-implementation surveys."*<br>**Page 9**<br>And: *"In cases of physiotherapy, occupational therapy, dietetics, speech pathology, social work, pharmacy, podiatry, orthotics and/or diversional therapy involvement, in the project team, verbal support from their respective manager is required."*<br>**Pages 7&8**<br>Table 2: Inclusion and exclusion criteria for components of the study was also updated to specify professions instead of the term "allied health professionals"
Please provide more information about the co-design. For example, what framework is being used, what steps will be followed, what techniques/methods (i.e., workshops, focus groups, design sprints, iterative prototyping), what is the decision-making process, etc.	Further information about the ward-level co-design of the multicomponent fall prevention interventions has been provided in the methods:<br>**Page 11**<br>*"The selection and co-design of specific interventions is informed by each ward's local fall incident data and the ward's unique*

	patient, organisational and environmental characteristics <u>and follows the Agency for Clinical Innovation's codesign framework (51).</u> <u>Ward teams attend workshops during the improvement science training and on the ward during the intensive implementation period where they brainstorm key causes of falls on their ward and potential management strategies (informed by staff and patient surveys, evidence updates and staff experiences).</u> This includes consideration of the cultural and linguistic backgrounds of the patient cohorts on their ward. <u>The ward teams work with an experienced clinical facilitator to prioritise, select and tailor interventions based on their anticipated impact and achievability. The number, timing and duration of workshops is dependent on ward needs."</u>
How were these implementation strategies selected? Are they co-designed? Do the key partners get an input about what implementation strategies have worked in their wards in the past and what haven't and what strategies they prefer/don't prefer? When describing the implementation strategies, please use the recommended frameworks/model for describing implementation strategies. These could be the Proctor's specification guidelines that include the following key elements: name of the strategy, actor, action, target, temporality, dose, and justification.	Thanks for your attention to the implementation strategy reporting in the protocol. We agree that it required revisiting to ensure optimal reporting.<br>We have replaced Table 3: Implementation strategies with a new, more detailed table that describes the implementation using Proctor's recommendations for specifying implementation strategies including name of the strategy, actor, action, target, temporality, dose, and justification. This updated table is provided at the end of this response document. To ensure adherence to formatting guidelines a more succinct 2-page version of this table included within the manuscript (**pages 12-14**). The complete table is included as supplementary material.<br><br>Some introduction text has been deleted as it is now included in this table.<br><br>We have also added an additional implementation strategy, (building a coalition) that the research team agreed was an important strategy employed but not adequately reported.
Related to implementation data, any data collected related to identifying which strategies work and which do not? How will you know how to fine-tune the implementation strategies?	Data collected in the staff surveys and semi-structured interviews aims to inform which implementation strategies do and don't work and to suggest changes to these. We have

	added text to the Data collection methods to make this clearer:<br>**Page 19**<br>"<u>*Free-text survey questions and specific questions in the interview guide intend to provide data informing the effectiveness and suitability of the individual implementation strategies and suggested future tailoring of these."*</u><br><br>The survey and interview guide are provided in the **supplementary material**. The staff interview guide in particular has a distinct section which intends to gather data on this topic:<br>**"***PART TWO: Implementation support*<br>*The next questions relate to the implementation (or support) strategies that were used to*<br>*support your ward to implement selected fall prevention interventions (or change ideas). The implementation strategies included:*<br>*• 2-day Improvement Science training course*<br>*• 4 months of weekly support from Sally (PROTECT Clinical Facilitator)*<br>*•Staff fall prevention education sessions*<br>*• Provision of a ward falls profile that summarised data from falls on your ward over the last 2 years and some patient and staff survey feedback.*<br>*• QIDS platform to monitor progress with change ideas*<br>*Referring to questions:2, 3, 4: We'll go through these implementation strategies one by one now and can you share what you found useful about them. And then anything you didn't like, or that didn't work about them. Additionally, if there is anything you'd change about them for the future."*
I am excited to see cost-effectiveness being included in this study and protocol as it is an essential aspect of both sustainability and scale-up. However, I also feel like it was just thrown in there/it appears to come out of nowhere on page 17. Thus, I recommend that either this piece is woven in more throughout the manuscript or removed from the manuscript. For example, there is no information about cost in the introduction. If cost-effectiveness is part of the study, then it is recommended that it is discussed from the	We have incorporated the plans for evaluations of the cost-effectiveness more consistently throughout the manuscript. We have added the following text to the introduction:<br>**Page 5**<br>"<u>*Economic evaluations are crucial to informing the cost-effectiveness, or value for money, of hospital fall prevention interventions, thus providing comprehensive evidence for decision-making in policy and practice, especially at scale (28). Guidelines*</u>

start. The methods also need to expanded upon. For example, whose perspective is this coming from (e.g., societal, healthcare system, payer, or patient). Is there a comparator? What costs are included? What are the outcome measures? Are you using CHEERS guidelines?	<u>*for economic evaluation of fall prevention strategies have been available since 2011(29). Despite this, few trials of interventions to reduce falls in hospital settings incorporate economic evaluations (7).”*</u><br><br>The introduction retains the line *“The cost-effectiveness of the program from a hospital perspective will also be evaluated.”*<br><br>The first two lines of the methods (page 5) specify that: *“This study is a stepped wedge (28) pragmatic hybrid type I effectiveness-implementation (29) cluster randomised trial. It has a nested economic evaluation.”*<br><br>The following information was provided in the data collection section of the methods. This section specifies that the cost-effectiveness analysis will include intervention and implementation cost data and healthcare utilisation data. We have made the subheading more distinct.<br>**Page 19**<br>***“Cost-effectiveness data collection***<br>*Health utilisation and intervention and implementation cost data*<br>*Intervention and implementation cost data for the economic evaluation will be collected from trial-based records <u>for the trial duration</u>. Healthcare utilisation during the trial duration will be collected from routinely collected coded hospital data. Healthcare utilisation will be valued using local or national costs as appropriate and all costs will be valued in Australian dollars. Discounting will not be applied, as the time horizon will be limited to the trial duration*.”<br><br>The following methods text more thoroughly describes the economic analysis plans. We have made this subheading more distinct. This section now specifies that the cost-effectiveness analysis is from a hospital perspective, the comparator in usual care, the outcome measures and the use of the CHEERS guidelines:<br>**Page 21**

	“***Economic analysis***<br>*A trial based economic evaluation will be conducted from a hospital perspective and the time horizon will be limited to the trial duration. The comparator will be usual care. All intervention implementation and delivery costs (from study records), including staff and equipment costs, and all health service utilisation costs (from hospital records) during the trial period (pre-implementation, implementation and follow-up periods) will be used for the analysis. We will calculate the incremental cost per fall prevented of the intervention periods compared with control periods.*<br><br>*Principles of economic evaluation alongside stepped wedge trials will be followed (60). Bootstrapping will estimate a distribution around costs and health outcomes and calculate the confidence intervals around the incremental cost effectiveness ratios. The results will be plotted on the cost-effectiveness plane. A cost-effectiveness acceptability curve will be created to explore the uncertainty around the probability that the intervention is cost-effective, when considering a decision maker’s willingness to pay for fall prevented. All analyses will be undertaken in Stata v16 (Stata Corp, College Station, TX). The results of the cost-effectiveness analysis will be reported following the CHEERS statement (61).”*<br><br>We intend to make a specific cost-effectiveness analysis protocol available prior to conducting the cost-effective analysis.

Supplementary material: Expanded table 2: Implementation strategies

**Core implementation strategy: Training (Quality improvement)**<br>**Branching implementation strategies:** Capture and share local knowledge; conduct local consensus discussion; conduct cyclical small tests of change; develop and implement tools for quality monitoring; identify and prepare champions; model and simulate change; tailor strategies; audit and provide feedback<br>**Action:** Improvement Science training course is run by the Local Health District’s education department. Quality improvement is a system of iterative review and refinement aiming to produce positive changes to optimise local and organisational outcomes[1].<br>Training topics include: using diagnostic data to inform change ideas; assessing and implementing change ideas’ via Plan-Do-See-Act (PDSA) cycles; and assessing, spreading and sustaining improvement. The course will use mixed modes of training including presentations,

workshops and interactive activities. The course will have 2 hours of additional hospital fall prevention evidence summary content added for the trial.					
**Actor**	**Target(s)**	**Temporality**	**Dose**	**Outcome affected**	**Justification**
This training is a pre-existing course available to staff across the four participating hospitals run by clinicians who are experts in quality improvement and fall prevention. The primary trainer is an Educator working for the health district's education department.	Minimum two clinicians from each participating ward who will be leading their team's implementation of multicomponent fall prevention interventions.	Staff attend the course within the two-week period prior to the commencement of the intensive clinical facilitation support	Once off, two-day face-to-face course.	Adoption, reach, dose, sustainability	Research suggests quality improvement training is acceptable and feasible in supporting the implementation of multicomponent fall prevention interventions[2, 3]. Face-to-face training is perceived to be more beneficial than online training[2].

**Core implementation strategy: Facilitation**<br>**Branching implementation strategies:** Distribute educational materials; organise clinician implementation team meetings; promote adaptability; remind clinicians; use train-the-trainer strategies; create a learning collaborative<br>**Action**: A Clinical Facilitator provides ongoing training and support to clinical staff on improvement science methodology following their two-day training course. The facilitator continues guidance for teams (including and beyond the team members who attended the training course) about how an improvement science approach can support tailored fall prevention interventions in their clinical areas, accommodating their working environment. Training and education may include topics such as using available data to inform and evaluate change; practical understanding of how to adapt ways of working to reflect their distinct ward/area setting, work culture, patient cohort and specific local challenges; implementing and managing change; and applying the theory of improvement science to falls prevention in a real-time clinical setting via PDSA cycles. The clinical facilitator can also provide the tailored ongoing fall prevention education and training described in the core implementation strategy below. Clinical facilitation is underpinned by theoretical models of behaviour change and involves an experienced clinician providing mentorship, guidance and support to clinical teams to support the uptake of positive changes in clinical practice[4].					
**Actor**	**Target(s)**	**Temporality**	**Dose**	**Outcome affected**	**Justification**
The Clinical Facilitator is	Staff on participating	Clinical facilitation	The clinical facilitator will	Adoption, reach, dose,	Clinical facilitation was

a full-time, trial-funded position filled by a senior Nurse Specialist. Their workload is distributed across the four participating wards at a time.	wards implementing multi-component fall prevention interventions.	commences in week one of the participating wards' 17-week intensive implementation support period.	meet face-to-face with the participating teams for at least 30minutes, once a week during the 17-week intensive implementation period and is available for access at other times on an as-needs basis. Teams then move to a minimum of 4 months of 30mins clinical facilitation per month (with additional support upon request).	fidelity, sustainability	successfully used to support multicomponent fall prevention intervention in a multi-site quality improvement project[3]. The team's prior research informing this study found clinical facilitation is acceptable and feasible in supporting the implementation of multicomponent fall prevention interventions[5].
**Core implementation strategy: Ongoing education and training**<br>**Branching implementation strategies:** Capture and share local knowledge; make training dynamic<br>**Action:** Tailored education about risks for and consequences of falling in hospital and training sessions on the practical delivery of ward-selected fall preventions strategies. Using adult learning techniques, staff education and training can increase staff engagement, skills, efficacy, confidence and motivation[6, 7].					
**Actor**	**Target(s)**	**Temporality**	**Dose**	**Outcome affected**	**Justification**
Education and training sessions are run by physiotherapy and nursing members of the study team experienced in hospital fall prevention and by the Clinical Facilitator.	Staff on participating wards implementing multi-component fall prevention interventions.	Education and training sessions occur during the intensive implementation phase	The specific content, number and timing of sessions is informed by ward staff needs and preferences identified in the staff pre-implementation surveys, in discussion with ward staff and in testing their selected	Adoption, reach, dose, fidelity	Staff education and training is an integral component of the delivery of effective fall prevention interventions in large trials in hospital settings[8, 9]. Staff education and training was identified as facilitator to multicomponent fall prevention

			small local changes.		in the team's prior research informing this study[2, 10].
**Core implementation strategy: Build a coalition**<br>**Action**: Internal and external stakeholders are engaged to optimise the implementation effort.<br>**Branching implementation strategies:** Identify and prepare champions; obtain and use patients/ consumers feedback; involve patients/consumers and family members; involve executive boards; promote network weaving; provide ongoing consultation; use advisory boards and workgroups; use an implementation advisor; work with educational institutions					
**Actor**	**Target(s)**	**Temporality**	**Dose**	**Outcome affected**	**Justification**
The research team intentionally map and engage stakeholders.	Internal and external stakeholders who will be involved, influence and/or impact the intervention and implementations strategies. Cohorts include but are not limited to academics, health executive and managers, clinicians, hospital staff, patients and families.	Commenced in months prior to study and in previous work informing the study. Continues throughout and after the study period.	The specific mode, number and timing of stakeholder interactions is dependent on stakeholder capacity and project needs.	Adoption, reach, dose, fidelity, sustainability.	The research team's relationships with broad stakeholder groups were crucial to the work informing this study[2, 10]. The implementation science field identifies a need for stakeholder engagement to optimise research and healthcare delivery[11].

REFERENCES

1. Puri I, Hollingshead, C, Tadi, P. . Quality Improvement. Treasure Island (FL) StatPEarls; 2023.
2. McLennan C, Sherrington C, Naganathan V, Tilden W, Richards B, McVeigh T, et al. Supported implementation of tailored multicomponent fall prevention interventions in hospital: a feasibility study. BMJ Open Qual. 2025;14(3).
3. Archer Y, Chasle B, Macpherson A, Wilson V. Advancing Quality and Safety Culture in Healthcare: Insights From the Evaluation of an Improvement Science Program for Nurses and Midwives. J Adv Nurs. 2025;81(6):3296-307.
4. Harvey G, Lynch E. Enabling Continuous Quality Improvement in Practice: The Role and Contribution of Facilitation. Front Public Health. 2017;5:27.
5. Curtis K, Fry M, Shaban RZ, Considine J. Translating research findings to clinical nursing practice. J Clin Nurs. 2017;26(5-6):862-72.

6. Shaw L, Kiegaldie D, Farlie MK. Education interventions for health professionals on falls prevention in health care settings: a 10-year scoping review. BMC Geriatr. 2020;20(1):460.
7. Fontaine G, Vinette B, Weight C, Maheu-Cadotte MA, Lavallee A, Deschenes MF, et al. Effects of implementation strategies on nursing practice and patient outcomes: a comprehensive systematic review and meta-analysis. Implement Sci. 2024;19(1):68.
8. Hill AM, McPhail SM, Waldron N, Etherton-Beer C, Ingram K, Flicker L, et al. Fall rates in hospital rehabilitation units after individualised patient and staff education programmes: a pragmatic, stepped-wedge, cluster-randomised controlled trial. Lancet. 2015;385(9987):2592-9.
9. Di Gennaro G, Chamitava L, Pertile P, Ambrosi E, Mosci D, Fila A, et al. A stepped-wedge randomised controlled trial to assess efficacy and cost-effectiveness of a care-bundle to prevent falls in older hospitalised patients. Age and Ageing. 2024;53(1).
10. McLennan C, Sherrington C, Tilden W, Jennings M, Richards B, Hill A-M, et al. Considerations across multiple stakeholder groups when implementing fall prevention programs in the acute hospital setting: a qualitative study. Age and Ageing. 2024;53(10).
11. Potthoff S, Finch T, Buhrmann L, Etzelmuller A, van Genugten CR, Girling M, et al. Towards an Implementation-STakeholder Engagement Model (I-STEM) for improving health and social care services. Health Expect. 2023;26(5):1997-2012.
